# Achiral organoiodine-functionalized helical polyisocyanides for multiple asymmetric dearomative oxidations

**DOI:** 10.1038/s41467-023-36327-0

**Published:** 2023-02-02

**Authors:** Zong-Quan Wu, Xue Song, Yan-Xiang Li, Li Zhou, Yuan-Yuan Zhu, Zheng Chen, Na Liu

**Affiliations:** 1 State Key Laboratory of Supramolecular Structureand Materials, College of Chemistry, Jilin University, Changchun, 130012 China; 2 Department of Polymer Science and Engineering, School of Chemistry and Chemical Engineering, Hefei University of Technology, Hefei, 230009 China; 3 The School of Pharmaceutical Sciences, Jilin University, 1266 Fujin Road, Changchun, Jilin 130021 China

**Keywords:** Asymmetric synthesis, Polymer synthesis, Catalyst synthesis

## Abstract

Immobilizing organocatalyst onto helical polymers not only facilitates the catalyst recycling from homogeneous reactions, but also boosts enantioselectivity. In this work, achiral organoiodine-functionalized single left- and right-handed helical polyisocyanides were prepared from the same monomers, which catalyzed three asymmetric oxidations gave the desired products in high yields and excellent enantioselectivity. The enantiomeric excess of the target products was up to 95%. Remarkably, the enantioselectivity can be switched by reversing the helicity of the polymer backbone. The polymer catalysts can be facilely recovered and recycled in different asymmetric oxidations with maintained excellent activity and enantioselectivity.

## Abstract (web summary)

Immobilizing organocatalysts onto helical polymers can facilitate catalyst recycling and boost enantioselectivity. Here, the authors prepare achiral organoiodine-functionalized single left- and right-handed helical polyisocyanides from the same monomers and demonstrate their use in three asymmetric oxidations.

## Introduction

Chemical catalysts are important for industrial applications and laboratory use. Considering the disadvantages of contemporary catalysts, catalytic systems are currently being further developed and improved. For example, homogeneous catalysts generally exhibiting a higher catalytic activity than comparable heterogeneous systems are more difficult to isolate, thereby often remaining in the final product^1–3^. In contrast, heterogeneous catalysts facilitate the separation and recycling processes, often with low activity and selectivity^4,5^. In recent years, catalyst recycling has increasingly become more important with regard to resource conservation and environmental protection^6,7^. Therefore, seeking an elegant balance between homogeneous and heterogeneous catalyzes is particularly significant^8,9^.

From a practical asymmetric synthesis point of view, soluble polymer-supported chiral catalysts are of great interest because of their ease of separation from the reaction mixture and their ability to facilitate recycling^10–15^. Polymer-supported chiral catalysts are generally obtained by embedding a well-established chiral molecular catalyst onto readily available achiral polymers^16–19^. Activity and selectivity are decreased in many cases with respect to the chiral molecular catalyst. In contrast, fabricating chiral polymer-supported catalysts by decorating an achiral molecular catalyst onto a chiral polymer skeleton has rarely been realized^20–22^. In this case, the polymer skeleton provides a chiral environment for the asymmetric reaction, while the catalyst moiety only serves as a catalytic site^23,24^. Thus, a variety of polymer-supported chiral catalysts can be facilely developed.

Biological systems apply homochirality (e.g., l-amino acids and d-sugars) and result in biological macromolecules adopting well-ordered helical structures like the right-handed *α*-helix in proteins and the right-handed double-strand helix in deoxyribonucleic acid (DNA)^25–27^. The high efficiency and stereospecificity of enzyme catalysis rely on the homochirality of the biomacromolecules^28,29^. Inspired by exquisite biological helices, an increasing number of artificial helical polymers has been developed^30–36^. These polymers exhibit a large optical activity solely due to the excess of a one-handed helicity^23,37–42^. Therefore, incorporating catalytic moieties onto helical polymers may establish novel polymer-supported chiral catalysts. This strategy has many advantages. For example, it provides a model system for elucidating the origin of natural homochirality and a path for developing chiral catalysts^43^. Moreover, enantioselectivity can be tuned through regulation on the polymer helicity. However, the lack of control over helical polymer structures prevents their applications in asymmetric reactions. We recently developed a series of alkyne-Pd(II) catalysts that promote living polymerizations of various isocyanides and give helical polyisocyanides with a predicted chain length, low dispersity, and controlled helicity^42,44–58^. The precisely synthesized helical polyisocyanides are a good chiral skeleton for catalyst supports. Chiral organoiodine-catalyzed asymmetric oxidation reactions have recently attracted great attention due to their mild reaction condition, low toxicity, and environmental friendliness^49–57^. However, owing to the low catalytic activity, the loading of molecular organoiodine catalysts is quite high and generally larger than 10 mol%. Furthermore, it is difficult to recycle small molecular catalysts from a homogeneous reaction solution. Thus, immobilizing organoiodine catalysts on helical polymers to boost enantioselectivity, facilitate catalyst separation, and recycling is greatly desirable. Immobilizing organoiodine onto rigid helical polyisocyanide has various superiorities. In addition to the facilitations on catalyst isolation and recycling, the unique rigid helical polyisocyanide facilitates the remote chirality transfer of helical backbone and renders the asymmetric reaction with high stereoselectivity. The high rigidity can effectively avoid the polymer chains entanglement and aggregation. Thus the asymmetric catalysis can be performed homogeneous and provides the polymer catalysts with high catalytic activity and efficiency. Owing to the high molar mass, the helical polyisocyanide-based catalysts can be readily recovered and recycled from the homogenous solution just by solvent precipitation.

In this work, a family of chiral polymer-supported catalysts is developed by immobilized achiral organoiodine catalyst onto one-handed helical polyisocyanides. First, left- and right-handed helical polyisocyanides (i.e., *M*-poly-L-**1**_150_ and *P*-poly-L-**1**_50_) in different molar masses are simultaneously prepared via the Pd(II)-catalyzed living polymerization of chiral isocyanide (L-**1**)^45,58^. The chain extension of these two polymers with organoiodine-functionalized achiral isocyanide (**2**) afford left-handed *M*-poly(L-**1**_150_-*b*-**2**_m_) and right-handed *P*-poly(L-**1**_50_-*b*-**2**_m_). These polymers can catalyze multiple asymmetric oxidations and give the desired products in acceptable yields and high enantioselectivity. Enantioselectivity is completely controlled by the backbone helicity. Although *M*-poly(L-**1**_150_-*b*-**2**_m_) and *P*-poly(L-**1**_50_-*b*-**2**_m_) are prepared from the same chiral materials, they produced enantiomeric products in similar yields but in opposite enantioselectivities. The enantiomeric excess (*ee*) of the generated product is up to 95% with 86% yield. The catalysts can be facilely separated from the homogeneous reaction mixture and recycled 10 times with maintained activity and selectivity. Remarkably, the polymer catalysts can successively catalyze three different asymmetric reactions with maintained high activity and enantioselectivity.

## Results

### Synthesis and characterization of achiral organoiodine-functionalized helical polyisocyanide catalysts

**Fig. 1:**
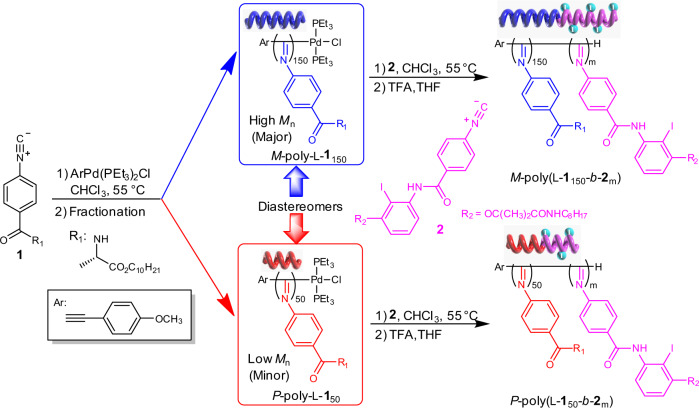
Catalysts synthesis. Synthesis of helical polyisocyanide-supported chiral catalysts.

**Fig. 2:**
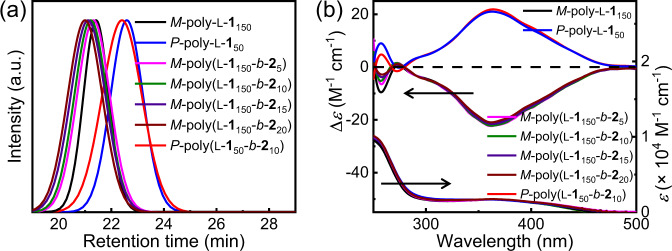
SEC traces, CD and UV–vis spectra of catalysts. Size exclusion chromatograms (**a**), and CD and UV–vis spectra (a.u. is the abbreviation of arbitrary units) (**b**) of the isolated single-handed helical polyisocyanides. The CD and UV–vis spectra were recorded in THF with 0.2 mg/mL at 25 °C.

Figure 1 shows that L-**1** was polymerized by the alkyne-Pd(II) catalyst in chloroform at 55 °C under a living manner ([L-**1**]_0_/[Pd]_0_ = 100). The polymerization simultaneously generated a left-handed *M*-poly-L-**1**_150_ with a high molar mass (*M*_n_) and a right-handed *P*-poly-L-**1**_50_ with a low *M*_n_, which were readily separated by solvent fractionation^44–48^. Supplementary Information (SI) presents further details (Supplementary Fig. 1-5). *M*_n_ and its dispersity (*M*_w_/*M*_n_) of *M*-poly-L-**1**_150_ were 57.7 kDa and 1.10, respectively, as measured by size exclusion chromatography (SEC) (Fig. 2a). *M*_n_ and *M*_w_/*M*_n_ of *P*-poly-L-**1**_50_ were 20.3 kDa and 1.14, respectively. The single-handed helicity of the two polymers was verified by circular dichroism (CD) analyses. Both *M*-poly-L-**1**_150_ and *P*-poly-L-**1**_50_ showed intense CD in the absorption region of the poly(phenyl isocyanide) backbone and was a mirror image of each other (Fig. 2b). The molecular CD intensity (Δ*ε*_1st_) at the first cotton band that reflected the backbone helicity was −21.02 M^−1^cm^−1^ for *M*-poly-L-**1**_150_ and +20.62 M^−1^cm^−1^ for *P*-poly-L-**1**_50_, confirming the single left- and right-handed helicities of the backbone, respectively^44–48^.

The isolated *M*-poly-L-**1**_150_ and *P*-poly-L-**1**_50_ carrying active Pd(II)-complex on the chain end are active for chain extension although the molar mass of the two polymers are quite different^45^. By virtue of the living chain end of the isolated polyisocyanides, a block copolymerization of iodine-functionalized monomer **2** with *M*-poly-L-**1**_150_ and *P*-poly-L-**1**_50_ was performed. The SEC profiles of the generated block copolymers shifted to a high-*M*_n_ region with respect to the precursors and maintained a mono-distribution (Fig. 2a). For instance, *M*_n_ and *M*_w_/*M*_n_ of the chain-extended *M*-poly(L-**1**_150_-*b*-**2**_10_) were 63.1 kDa and 1.15, respectively. Similarly, the block copolymerization of **2** with *P*-poly-L-**1**_50_ gave *P*-poly(L-**1**_50_-*b*-**2**_10_) with *M*_n_ = 25.3 kDa and *M*_*w*_/*M*_*n*_ = 1.18 (Fig. 2a). For comparison, homopolymer poly-**2**_20_ was prepared via the living polymerization of **2** by the alkyne-Pd(II) catalyst. The structures of these polymers were further confirmed by ^1^H NMR and Fourier transform infrared spectroscopy (FT-IR) spectra together with SEC analyses (Supplementary Figs. 6–15 and Supplementary Table 1). ^1^H NMR of the block copolymer showed characteristic signals at 3.60–3.05 ppm corresponding to the methylene protons of the poly-**2**_m_ segment. FT-IR of the block copolymers exhibited the characteristic vibrations of the C=N, OC=O, and NHC=O bonds at 1600, 1626, and 1750 cm^−1^, respectively, which confirmed the formation of the expected block copolymers (Supplementary Figs. 6–15).

The molecular CD profiles of the block copolymers were almost similar to their macroinitiators, suggesting that the helicity of the newly formed poly-**2**_m_ segments adopted the same helical structures to the precursors (Fig. 2b). The Δ*ɛ*_1st_ was −22.18 M^−1^cm^−1^ for *M*-poly(L-**1**_150_-*b*-**2**_10_) and +21.87 M^−1^cm^−1^ for *P*-poly(L-**1**_50_-*b*-**2**_10_). The analyses confirmed the single-handed helicity of the whole backbone of the block copolymers^44–48^. No other chiral moiety was involved in the poly-**2**_m_ block; hence, the optical activity of the poly-**2**_m_ segment in the block copolymers was solely from the one-handed helical backbone. Note that the helicity was quite stable. Both *M*-poly(L-**1**_150_-*b*-**2**_10_) and *P*-poly(L-**1**_50_-*b*-**2**_10_) could maintain their CD and absorption profiles at a temperature ranging from −30 °C to 50 °C in tetrahydrofuran (THF) (Supplementary Fig. 16a). Moreover, the helical structures were stable in various organic solvents, regardless of the polarity (e.g., THF, toluene, dichloromethane, and chloroform) (Supplementary Fig. 16b).

### Catalytic performance of catalysts in Kita-Spirocyclization

To investigate the catalytic activity of the synthetic polymers, an achiral poly-**2**_20_ homopolymer was prepared and used to catalyze the Kita spirocyclization of 1-naphthol carboxylic acid **3**^55^. The reaction was conducted in dichloromethane (CH_2_Cl_2_) at 0 °C with 5 mol% catalyst loading in the presence of *m*-chloroperoxybenzoic acid (*m*CPBA, 0.03 mmol) as the oxidant. As a result, it provided the targeted product in 80% yield, but without enantioselectivity (run 1 in Table 1) because of the inexistence of chirality in the catalyst and substrate. Meanwhile, *M*-poly-L-**1**_150_ could not catalyze the reaction due to the inexistence of an organoiodine moiety (run 2 in Table 1). The catalytic performance of the helical polymer-supported catalyst *M*-poly(L-**1**_150_-*b*-**2**_5_) on the spirocyclization of **3** was then conducted^59,60^. As anticipated, the target product **4** **s** was presented in 75% yield (run 3 in Table 1). The enantiomeric excess (*ee*) of **4s** was 62%, as determined by chiral high-performance liquid chromatography (HPLC). With the increased content of the poly-**2**_m_ segment, *M*-poly(L-**1**_150_-*b*-**2**_10_) gave the same product in 79% yield and 62% *ee*, suggesting that the organoiodine content slightly affected the reaction (runs 3–4 in Table 1, and Supplementary Table 2). The *M*-poly(L-**1**_150_-*b*-**2**_15_) and *M*-poly(L-**1**_150_-*b*-**2**_20_) with longer poly-**2**_m_ segment gave **4** **s** in 78% yields with *ee* decreased to 59% and 57%, respectively (runs 5–6 in Table 1). Because inexistence of chiral pendants, the longer poly-**2**_m_ could not fully maintaine the one-handed helicity, and thus the enantioselectivity was decreased. Detailed study revealed that the helicity of *M*- and *P*-poly-**1**_m_ blocks could be transfer to poly-**2**_m_ with degree of the polymerization of 20. Further increase the length of poly-**2**_m_ segment, it could not effectively maintain the one-handed helicity, as revealed by CD and UV-vis analyses (Supplementary Fig. 17). Thus, *M*-poly(L-**1**_150_-*b*-**2**_10_) was used in the subsequent studies. The helicity of the organoiodine-immobilized helical polymers was quite stable; hence, the spirocyclization of **3** catalyzed by *M*-poly(L-**1**_150_-*b*-**2**_10_) was performed in various solvents, including chloroform, THF, toluene, and chlorobenzene (runs 7–10 in Table 2). Consequently, chloroform showed the best performance, giving **4s** in 83% yield and 72% *ee* (run 7 in Table 1). A further attempt of optimizing the condition using ethanol (EtOH) and methanol (MeOH) as additives resulted in an increased yield, but decreased *ee* (runs 11, 12 in Table 1). Lowering the reaction temperature further improved the enantioselectivity. For example, conducting the reaction at ‒30 °C gave **4s** in 87% yield and 95% *ee*. Further lowering the temperature caused the catalyst precipitation. *M*-poly(L-**1**_150_-*b*-**2**_10_) contained chiral L-**1** moieties and a left-handed helical backbone; thus, to disclose the origination of the enantioselectivity, a right-handed helical *P*-poly(L-**1**_50_-*b*-**2**_10_) was used to catalyze the same spirocyclization of **3**. Interestingly, the enantiomeric product **4r** in 85% yield and −93% *ee* was obtained. Note that *M*-poly(L-**1**_150_-*b*-**2**_10_) and *P*-poly(L-**1**_50_-*b*-**2**_10_) were prepared from the same monomers, but had opposite backbone helicities. The opposite enantioselectivity of the reaction was ascribed to the helical backbone of the polymer catalysts. These results demonstrate that the enantioselectivity in asymmetric reactions completely depends on the helical backbone, not on the chiral pendants of L-**1**. Therefore, enantiomeric products could be facilely produced from the same chiral source mediated by helical polymers.

**Table 1:** Kita-Spirocyclization of 3^a^


Run	Catalyst	Solvent	Temp.	Yield^b^	*ee*^c^
1	Poly-**2**_20_	CH_2_Cl_2_	0 °C	80%	0
2	*M*-poly-L-**1**_150_	CH_2_Cl_2_	0 °C	n.d.^d^	n.d.^d^
3	*M*-poly(L-**1**_150_-*b*-**2**_5_)	CH_2_Cl_2_	0 °C	75%	62%
4	*M*-poly(L-**1**_150_-*b*-**2**_10_)	CH_2_Cl_2_	0 °C	79%	62%
5	*M*-poly(L-**1**_150_-*b*-**2**_15_)	CH_2_Cl_2_	0 °C	78%	59%
6	*M*-poly(L-**1**_150_-*b*-**2**_20_)	CH_2_Cl_2_	0 °C	78%	57%
7	*M*-poly(L-**1**_150_-*b*-**2**_10_)	CHCl_3_	0 °C	83%	72%
8	*M*-poly(L-**1**_150_-*b*-**2**_10_)	THF	0 °C	63%	41%
9	*M*-poly(L-**1**_150_-*b*-**2**_10_)	toluene	0 °C	62%	56%
10	*M*-poly(L-**1**_150_-*b*-**2**_10_)	PhCl	0 °C	55%	52%
11^e^	*M*-poly(L-**1**_150_-*b*-**2**_10_)	CHCl_3_	0 °C	87%	66%
12^f^	*M*-poly(L-**1**_150_-*b*-**2**_10_)	CHCl_3_	0 °C	86%	68%
13	*M*-poly(L-**1**_150_-*b*-**2**_10_)	CHCl_3_	‒30 °C	87%	95%
14	*P*-poly(L-**1**_50_-*b*-**2**_10_)	CHCl_3_	‒30 °C	85%	−93%

^a^Unless otherwise denoted, all reactions were carried out with **3** (0.02 mmol), catalyst (5 mol% of the phenyl iodine pendants), and *m*CPBA (0.03 mmol) in specific solvent (2.0 mL) at different temperature (temp.).

^b^Yield of isolated products.

^c^The *ee* values are referred to the major isomer determined by HPLC using a chiral stationary phase.

^d^Not detected.

^e^Using 20 eq. ethanol as additive.

^f^Using 20 eq. methanol as additive.

**Table 2:** Dearomatizative Spirocyclization of 5^a^


Run	Catalyst	Solvent	Temp.	Yield^b^	*ee*^c^
1	Poly-**2**_20_	MeNO_2_	0 °C	62%	0
2	*M*-poly-L-**1**_150_	MeNO_2_	0 °C	n.d.^d^	n.d.^d^
3	*M*-poly(L-**1**_150_-*b*-**2**_5_)	MeNO_2_	0 °C	52%	33%
4	*M*-poly(L-**1**_150_-*b*-**2**_10_)	MeNO_2_	0 °C	56%	33%
5	*M*-poly(L-**1**_150_-*b*-**2**_15_)	MeNO_2_	0 °C	56%	30%
6	*M*-poly(L-**1**_150_-*b*-**2**_20_)	MeNO_2_	0 °C	57%	27%
7	*M*-poly(L-**1**_150_-*b*-**2**_10_)	CH_2_Cl_2_	0 °C	27%	69%
8	*M*-poly(L-**1**_150_-*b*-**2**_10_)	CHCl_3_	0 °C	64%	68%
9	*M*-poly(L-**1**_150_-*b*-**2**_10_)	toluene	0 °C	33%	44%
10	*M*-poly(L-**1**_150_-*b*-**2**_10_)	THF	0 °C	n.d.^d^	n.d.^d^
11	*M*-poly(L-**1**_150_-*b*-**2**_10_)	1,4-dioxane	0 °C	n.d.^d^	n.d.^d^
12	*M*-poly(L-**1**_150_-*b*-**2**_10_)	acetone	0 °C	46%	39%
13^e^	*M*-poly(L-**1**_150_-*b*-**2**_10_)	CHCl_3_/THF	‒30 °C	65%	91%
14^e^	*P*-poly(L-**1**_50_-*b*-**2**_10_)	CHCl_3_/THF	‒30 °C	64%	−90%

^a^Unless otherwise denoted, all reactions were carried out using **5** (0.02 mmol), catalyst (5 mol% of the phenyl iodine pendants), *m*CPBA (0.03 mmol), 2,2,2-trifluoroethanol (TFE, 0.4 mmol), and H_2_O (0.16 mmol) in specific solvent (1.0 mL).

^b^Yield of isolated products.

^c^The *ee* values are referred to the major isomer determined by HPLC analysis using a chiral stationary phase.

^d^Not detected.

^e^CHCl_3_/THF = 5/1 (v/v).

### The recovery and recycling of catalysts

**Fig. 3:**
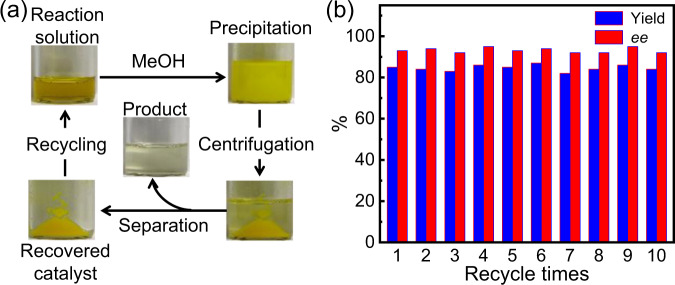
Recycle diagram and result statistics of catalysts. **a** Process of the catalyst recovery and recycling. **b** Yields and *ee* values of the products obtained from the asymmetric reaction of **3** catalyzed by recycled *M*-poly(L-**1**_150_-*b*-**2**_10_).

The block copolymer catalysts are solvable in chloroform, dichloromethane, toluene, and THF, but insoluble in acetone, methanol, and *n*-hexane. These features provide them with great potential in catalyzing asymmetric reactions in homogeneous catalysts and transferring into heterogeneous catalysts by adding poor solvents after the reaction is accomplished. Thus, we explored herein the catalyst separation and recycling, which are major advantages of polymer-supported catalysts. In Fig. 3a, the reaction of **3** catalyzed by *M*-poly(L-**1**_150_-*b*-**2**_10_) in CHCl_3_ was homogeneous all throughout the whole reaction process, even if the reaction was performed at −30 °C. However, the solution became heterogeneous after methanol or other poor solvent (e.g., *n*-hexane) was added. The high-*M*_n_ *M*-poly(L-**1**_150_-*b*-**2**_10_) was precipitated from the reaction solution, while the generated product, unreacted substrate, and other additives were dissolved (Fig. 3a). After centrifugation and filtration, the polymer-supported catalyst *M*-poly(L-**1**_150_-*b*-**2**_10_) was facilely recovered and isolated in an almost quantitative yield (Supplementary Fig. 18-20). The recovered *M*-poly(L-**1**_150_-*b*-**2**_10_) was reused to catalyze the spirocyclization of **3** again under the abovementioned condition and verify the catalytic activity. The reaction gave the desired product **4** **s** in 85% yield and 93% *ee*, suggesting that the recovered *M*-poly(L-**1**_150_-*b*-**2**_10_) catalyst maintained excellent activity and enantioselectivity. The catalyst was recycled 10 times without significant losses on selectivity and activity (Fig. 3b, and Supplementary Table 3).

### Catalytic performance of catalysts in others asymmetric oxidations

We investigated another asymmetric oxidative spirocyclization of 1-hydroxy-*N*-methyl-*N*-phenyl-2-naphthamide **5** (Table 2) to prove the general activity of this helical polymer-supported chiral catalyst^60^. For comparison, poly-**2**_20_ and *M*-poly-L-**1**_150_ were first used in this reaction. As expected, achiral poly-**2**_20_ generated the target product **6** in 62% yield but without enantioselectivity (run 1 in Table 2). Meanwhile, *M*-poly-L-**1**_150_ could not catalyze this reaction because of the lack of organoiodine pendants (run 2 in Table 2). *M*-poly(L-**1**_150_-*b*-**2**_10_) that catalyzed the dearomatizing spirolactonization of **5** gave the target product **6r** in 56% yield and 33% *ee* (run 4 in Table 2). Accordingly, the polymer composition and the reaction solvent were screened (runs 7–12 in Table 2), thanks to the high stability of the helical structure in various solvents. In terms of the yield and the *ee* of the product, CHCl_3_ showed the best performance. The *ee* and the yield of the afforded **6r** using *M*-poly(L-**1**_150_-*b*-**2**_10_) as catalyst were 64% and 68%, respectively (run 8 in Table 2). Interestingly, the *ee* was enhanced to 91% when the reaction temperature was lowered to −30 °C in CHCl_3_, and the yield was 65% (run 13 in Table 2). The corresponding enantiomeric product **6s** was obtained in ‒90% *ee* and 64% yield when the right-handed helical copolymer *P*-poly(L-**1**_50_-*b*-**2**_10_) was used (run 14 in Table 2). These results once again showed that the asymmetric selectivity was controlled by the helical backbone of the polyisocyanides. The helical polymer-supported catalyst can also be recovered and recycled for 10 times at least, and the catalytic activity and the enantioselectivity were generally maintained (Supplementary Fig. 21 and Supplementary Table 4).

As anticipated, poly-**2**_20_ catalyzed the oxidation of 2-naphthol carboxylic acid (**7**) without enantioselectivity, and *M*-poly-L-**1**_150_ had no catalytic activity on this reaction (runs 1, 2 in Table 3)^61^. However, *M*-poly(L-**1**_150_-*b*-**2**_m_) composed the same poly-L-**1**_100_, and the poly-**2**_m_ blocks showed an excellent performance on the oxidation reaction of **7** and yielded the desired product **8** **s** in 75% yield and 57% *ee* (runs 1–3 in Table 3). The reaction condition optimization revealed that the best result could be obtained in CH_2_Cl_2_ at −20 °C using *M*-poly(L-**1**_150_-*b*-**2**_10_) as a catalyst (runs 3–11 in Table 3). In this case, target **8** **s** was produced in 83% yield and 86% *ee* (run 11 in Table 3). When *P*-poly(L-**1**_50_-*b*-**2**_10_) with opposite handedness was used, the enantiomeric **8r** was obtained in 82% yield and −83% *ee* (run 12 in Table 3), further supporting that the reaction enantioselectivity was determined by the polyisocyanide backbone helicity.

**Table 3:** Dearomatizative spirocyclization of 7^a^


Run	Catalyst	Solvent	Temp.	Yield^b^	*ee*^c^
1	Poly-**2**_20_	CH_2_Cl_2_	0 °C	77%	0
2	*M*-poly-L-**1**_150_	CH_2_Cl_2_	0 °C	n.d.^d^	n.d.^d^
3	*M*-poly(L-**1**_150_-*b*-**2**_5_)	CH_2_Cl_2_	0 °C	75%	57%
4	*M*-poly(L-**1**_150_-*b*-**2**_10_)	CH_2_Cl_2_	0 °C	76%	57%
5	*M*-poly(L-**1**_150_-*b*-**2**_15_)	CH_2_Cl_2_	0 °C	78%	53%
6	*M*-poly(L-**1**_150_-*b*-**2**_20_)	CH_2_Cl_2_	0 °C	78%	51%
7	*M*-poly(L-**1**_150_-*b*-**2**_10_)	CHCl_3_	0 °C	71%	52%
8	*M*-poly(L-**1**_150_-*b*-**2**_10_)	toluene	0 °C	60%	44%
9	*M*-poly(L-**1**_150_-*b*-**2**_10_)	THF	0 °C	61%	31%
10^e^	*M*-poly(L-**1**_150_-*b*-**2**_10_)	CH_2_Cl_2_	0 °C	81%	71%
11^e^	*M*-poly(L-**1**_150_-*b*-**2**_10_)	CH_2_Cl_2_	‒20 °C	83%	86%
12^e^	*P*-poly(L-**1**_50_-*b*-**2**_10_)	CH_2_Cl_2_	‒20 °C	82%	−83%

^a^Unless otherwise denoted, all reactions were carried out using **7** (0.02 mmol), catalyst (5 mol% of the phenyl iodine pendants), and *m*CPBA (0.03 mmol) in specific solvent (2.0 mL).

^b^Yield of isolated product.

^c^The *ee* values are referred to the major isomer determined by HPLC analysis using a chiral stationary phase.

^d^Not detected (n.d.).

^e^Using 20 equiv. of hexafluoroisopropanol (HFIP) as additive.

**Fig. 4:**
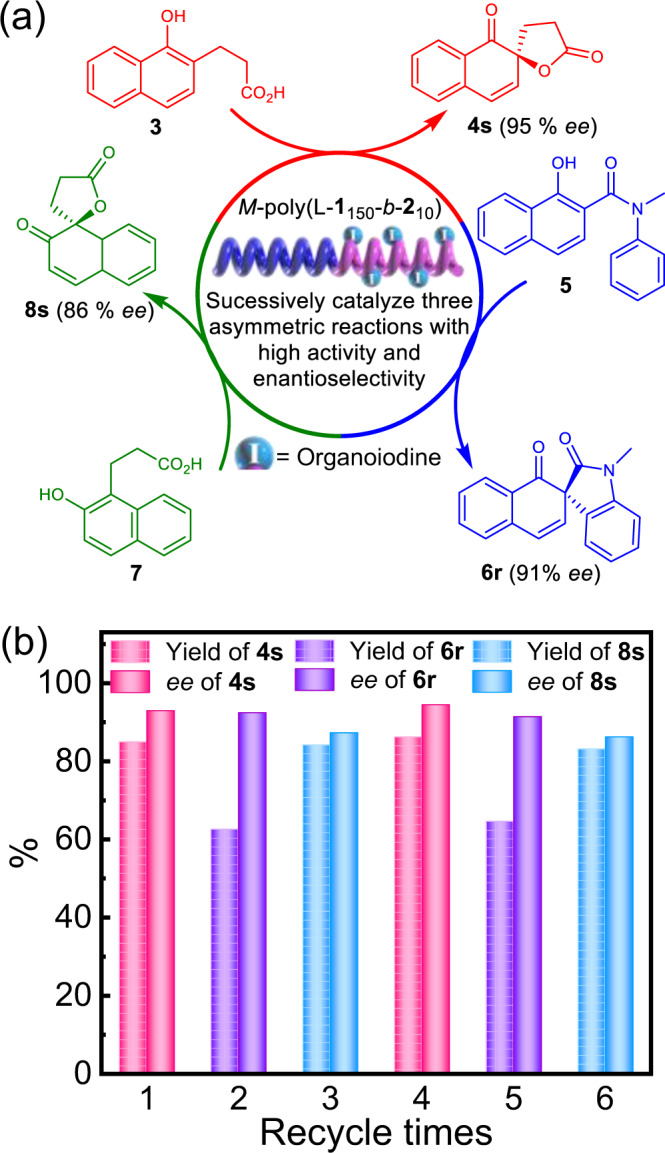
Cartoon diagram and results of catalyst cross catalyzing multiple reactions. **a** Recycling and successively catalyzed three different asymmetric reactions of **3**, **5**, and **7** using *M*-poly(L-**1**_150_-*b*-**2**_10_) catalyst. **b** Yields and *ee* values of **4s**, **6r**, and **8s**, obtained from the asymmetric reactions of **3**, **5**, and **7** catalyzed by recycled *M*-poly(L-**1**_150_-*b*-**2**_10_).

Lastly, we investigated the utilization of a single helical polymer-supported catalyst to promote three different asymmetric reactions. Figure 4a depicts that the recovered *M*-poly(L-**1**_150_-*b*-**2**_10_) from the reaction of **3** was directly used to catalyze the reaction of **5**, giving the expected product **6r** in 61% yield and 90% *ee*. After the reaction, *M*-poly(L-**1**_150_-*b*-**2**_10_) was recovered and reused to catalyze the reaction of **7**. It generated the desired **8s** in 82% yield and 85% *ee*. *M*-poly(L-**1**_150_-*b*-**2**_10_) was recycled six times for the three different asymmetric oxidations with high reactivity and enantioselectivity (Fig. 4b, and Supplemenatry Table 5).

## Discussion

### Mechanism study

**Fig. 5:**
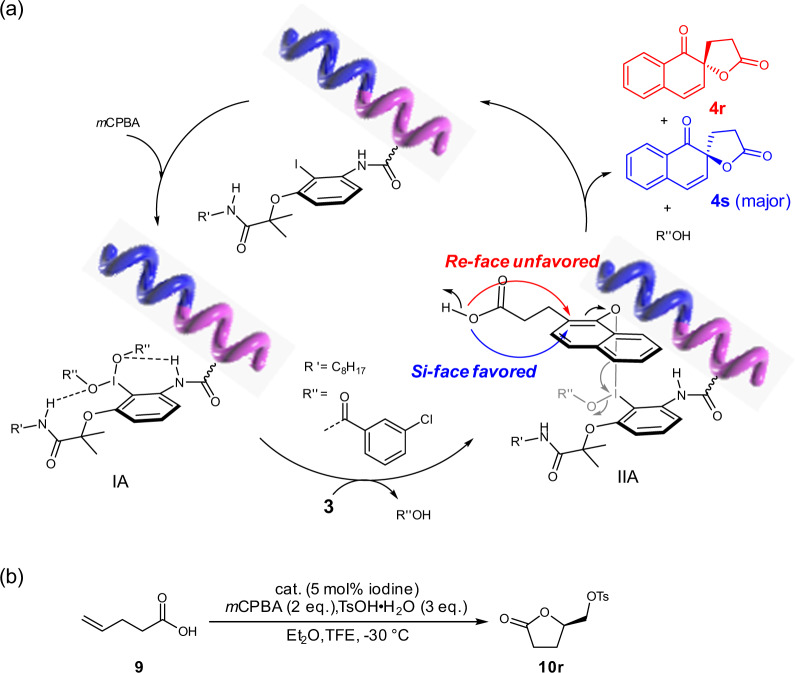
Mechanism study and asymmetric sulfonyl-oxylactonization. **a** Proposed mechanism for asymmetric Kita-Spirocyclization catalyzed by helical polyisocyanides. **b** Asymmetric sulfonyl-oxylactonization catalyzed *M*-poly(L-**1**_150_-*b*-**2**_10_).

A mechanism for the helical polyisocyanide catalyzed asymmetric reactions was proposed (Fig. 5a)^62–64^. Taking the asymmetric Kita-Spirocyclization as an example, the phenyl iodine pendants of polyisocyanide were initially oxidized to active hypervalent iodane (IA) by *m*CPBA. Then, the acetate on the hypervalent iodine was replaced by the hydroxyl of substrate **3**. In the resulting intermediate (IIA), the attack of carboxylic acid to the *ipso* position of naphthol can be happened from *Re*- or *Si*-face and lead to **4r** or **4s**, respectively. Owing to the asymmetric environment provided by remote chirality transfer of helical polyisocyanide, the carboxylic acid attack was selectively occurred on the favored *Si*-face with less steric hindrance, thus gave the target **4r** in good yield with high *ee* value. Other asymmetric reactions catalyzed by the iodine-functionalized polyisocyanides may proceed under mechanism similar to that of Kita-Spirocyclization due to the chiral environments induced by the one-handed helix.

Owing to the effectively remote chirality transfer of the rigid helical polyisocyanide backbone, the organoiodine-functionalized polyisocyanides also showed high performance on a more challenging intermolecular asymmetric sulfonyl-oxylactonization reaction (Fig. 5b). For example, *M*-poly(L-**1**_150_-*b*-**2**_10_) catalyzed the reaction of 4-pentenoic acid with *p*-toluenesulfonic acid in ethyl ether at −30 °C with the presence of TFE and *m*CPBA, gave the target product (5-oxotetrahydrofuran-2-yl)methyl 4-methylbenzenesulfonate (**10r**) in 72% yield with 74% *ee* (run 7 in Supplementary Table 6). This result suggests the organoiodine-functionalized helical polyisocyanides have great potentials in various asymmetric catalysis.

In summary, this work facilely obtained single left- and right-handed helical polyisocyanides immobilized with achiral organoiodine catalysts. These polymers exhibited a large optical activity and had a high helical stability. Interestingly, the helical polymer-supported catalysts showed an excellent performance on asymmetric oxidations. The product *ee* was up to 95%. Although the left- and right-handed helical polymers were prepared from the same materials, they exhibited opposite enantioselectivities on the asymmetric reactions. The enantioselectivity can readily be switched through regulation on the helix-sense of the polymer backbone. The catalyst can be facilely recycled and successively catalyze three different asymmetric reactions with excellent activity and enantioselectivity. This study provides not only a series of excellent polymer-supported chiral organocatalysts, but also a clue for the design and synthesis of advanced catalysts for asymmetric reactions.

## Methods

### Synthesis of M-poly-L-1_150_ and P-poly-L-1_50_

These polymers were prepared according to the reported procedure^44,45^. Monomer L**-1** (600 mg, 1.68 mmol) and alkyne-Pd(II)-catalyst (8.55 mg, 0.0168 mmol) were dissolved in dry CHCl_3_ (7.5 mL). The solution was stirred for 8 h at 55 °C, then cooled to ambient temperature and precipitated by methanol. The precipitated polymer was collected and dried in vacuum. The afforded crude polymer was suspended in acetone (180 mL) for 3 h with stirring and was filtrated. The filtrate was dried by evaporation under reduced pressure, afforded the expected *P*-poly-L-**1**_50_ (52 mg, 11% yield). SEC: *M*_n_ = 20.3 kDa, *M*_w_/*M*_n_ = 1.14; ^1^H NMR (600 MHz, CDCl_3_, 25 °C): *δ* 9.34–7.89 (br, NH), 7.10–5.47 (br, ArH), 5.05–3.65 (br, CH and CH_2_), 2.12–0.61 (br, CH_2_ and CH_3_); FT-IR (KBr, cm^−1^): *ν* 2919 (*ν*_C–H_), 2849 (*ν*_C–H_), 1744 (*ν*_NHC=O_), 1633 (*ν*_OC=O_), 1604 (*ν*_C=N_); [α]^25^_D_ = 1788 (0.1, CHCl_3_). The filter cake was dissolved in CHCl_3_ (6 mL) and then precipitated in acetone. The precipitate solid was collected by filtration and dried in vacuum. After this procedure was repeated 3 times, the left-handed *M*-poly-L-**1**_150_ was obtained (339 mg, 60%). SEC: *M*_n_ = 57.7 kDa, *M*_w_/*M*_n_ = 1.10; ^1^H NMR (600 MHz, CDCl_3_, 25 °C): *δ* 9.24–8.00 (br, NH), 7.16–5.70 (br, ArH), 4.88–3.50 (br, CH and CH_2_), 1.95–0.45 (br, CH_2_ and CH_3_); FT-IR (KBr, cm^−1^): *ν* 2925 (*ν*_C–H_), 2857 (*ν*_C–H_), 1751 (*ν*_NHC=O_), 1636 (*ν*_OC=O_), 1600 (*ν*_C=N_); [α]^25^_D_ = –1820 (0.1, CHCl_3_).

### Synthesis of M-poly(L-1_150_-b-2_m_) and P- poly(L-1_50_-b-2_m_)

Taking *M*-poly(L-**1**_150_-*b*-**2**_10_) as an example, the solution of **2** (8.13 mg, 0.014 mmol) and *M*-poly-L-**1**_150_ (50 mg) in CHCl_3_ (1.0 mL) was stirred for 10 h at 55 °C, then cooled to 25 °C and precipitated in acetone. After centrifugation, the polymer was collected and dried in vacuum, which was re-dissolved in CH_2_Cl_2_ (1 mL) and cooled to 0 °C. To this solution, trifluoroacetic acid (22 μL, 0.3 mmol) was added. The mixture was warmed to 25 °C and stirred for 12 h. Then, the solution was precipitated into methanol, and the precipitated polymer was collected and dried to afford a yellow solid *M*-poly(L-**1**_150_-*b*-**2**_10_) (46 mg, 80% yield). SEC: *M*_n_ = 63.1 kDa, *M*_w_/*M*_n_ = 1.15. ^1^H NMR (600 MHz, CDCl_3_, 25 °C): *δ* 8.95–8.15 (br, NH), 7.45–4.80 (br, ArH), 4.93–3.78 (br, CH and CH_2_), 3.64–2.89 (br, CH_2_), 2.02–0.65 (br, CH_2_ and CH_3_); FT-IR (KBr, cm^−1^): *ν* 2921 (*ν*_C–H_), 2853 (*ν*_C–H_), 1750 (*ν*_NHC=O_), 1626 (*ν*_OC=O_), 1600 (*ν*_C=N_); [α]^25^_D_ = –1906 (0.1, CHCl_3_). *P*-poly(L-**1**_50_-*b*-**2**_10_) was prepared followed the similar procedure in 82% yield by using *P*-poly-L-**1**_50_ as macro initiator. SEC: *M*_n_ = 25.3 kDa, *M*_w_/*M*_n_ = 1.18; ^1^H NMR (600 MHz, CDCl_3_, 25 °C): *δ* 8.87–8.13 (br, NH), 7.67–5.35 (br, ArH), 5.02–3.75 (br, CH and CH_2_), 3.58–2.95 (br, CH_2_), 2.63–0.66 (br, CH_2_ and CH_3_); FT-IR (KBr, cm^−1^): *ν* 2929 (*ν*_C–H_), 2852 (*ν*_C–H_), 1749 (*ν*_NHC=O_), 1630 (*ν*_OC=O_), 1609 (*ν*_C=N_); [α]^25^_D_ = 1894 (0.1, CHCl_3_).

### Typical procedure for asymmetric reaction

Taking the reaction of **3** as an example (see Supplementary Information for the procedure of **5** and **7**). The compound **3** (4.28 mg, 0.02 mmol) was dissolved in CHCl_3_ (2.0 mL) at 0 °C. Then *M*-poly(L-**1**_150_-*b*-**2**_10_) (5 mol% of the phenyl iodine pendants) and *m*CPBA (4.47 mg, 0.03 mmol) were consecutively added. The mixture was stirred at ‒30 °C for 3 days, then saturated Na_2_S_2_O_3_ and 1 M aq. Na_2_CO_3_ were added. The aqueous layer was extracted with ethyl acetate and the combined organic layers were washed with brine and dried over Na_2_SO_4_. After evaporation, the crude product was purified by column chromatography on silica gel (petroleum ether/ethyl acetate, v/v = 5/1) to afford compound **4** as a white solid. ^1^H NMR (600 MHz, CDCl_3_, 25 °C): *δ* 8.02 (d, *J* = 6.8 Hz, 1H), 7.63 (td, *J* = 7.6, 1.4 Hz, 1H), 7.41 (td, *J* = 7.6, 1.2 Hz, 1H), 7.26 (d, *J* = 12.0 Hz, 1H), 6.66 (d, *J* = 9.8 Hz, 1H), 6.21 (d, *J* = 9.8 Hz, 1H), 2.91 (ddd, *J* = 17.6, 11.2, 9.6 Hz, 1H), 2.60 (ddd, *J* = 17.6, 9.6, 2.2 Hz, 1H), 2.42 (ddd, *J* = 13.6, 9.6, 2.2 Hz, 1H), 2.21–2.16 (m, 1H).

## Supplementary information

Supplementary Information

## Data Availability

The synthetic details and experimental data generated in this study are provided in the Supplementary Information.
