# Supplementary material for: Achiral organoiodine-functionalized helical polyisocyanides for multiple asymmetric dearomative oxidations
Source: Nat Commun. 2023 Feb 2;14:566. doi: 10.1038/s41467-023-36327-0 (PMC9894859; doi:10.1038/s41467-023-36327-0)
Supplement: Supplementary file 1 — Supplementary Information [file 41467_2023_36327_MOESM1_ESM.pdf]

Supplementary Information

Achiral Organoiodine-Functionalized Helical Polyisocyanides for Multiple Asymmetric Dearomative Oxidations

Zong-Quan Wu,[1,] Xue Song,[2] Yan-Xiang Li,[2] Li Zhou,[2] Yuan-Yuan Zhu,[2] Zheng Chen,[1] and Na Liu[3]*

[1]State Key Laboratory of Supramolecular Structure and Materials, College of Chemistry, Jilin University, Changchun 130012, China

[2]Department of Polymer Science and Engineering, Hefei University of Technology, Hefei 230009, China

[3]The School of Pharmaceutical Sciences, Jilin University, 1266 Fujin Road, Changchun, Jilin 130021, P.R. China.

Supplementary Methods S3

Measurements S3

Materials S3

Supplementary Discussion S4

Synthesis of **2** and Supplementary Fig. 1..S4-S7

Polymerization procedure S7-S9

Procedures for asymmetric catalysis and Supplementary Fig. 2-5.................. S9-S11

Supplementary Notes..S12

Supplementary Table 1. Characterization for polyisocyanides..............................S12

Supplementary Table 2. The effect of catalyst loading on the reaction.................S12

Supplementary Table 3. Recycling *M*-poly(L-$\mathbf{1}_{150}$-*b*-$\mathbf{2}_{10}$) on the reaction of **3**......S13

Supplementary Table 4. Recycling *M*-poly(L-$\mathbf{1}_{150}$-*b*-$\mathbf{2}_{10}$) on the reaction of **5**......S13

Supplementary Table 5. Recycling *M*-poly(L-$\mathbf{1}_{150}$-*b*-$\mathbf{2}_{10}$) on **3**, **5**, and **7**................S14

Supplementary Table 6. Optimization of sulfonyloxylactonization of **9**..............S13

Supplementary Fig. 6-15. ^{1}H NMR and FT-IR of polyisocyanides............... S15-S15

Supplementary Fig 16-17. CD and UV-vis of polyisocyanides.............................S20

Supplementary Fig. 18-21. Characterization for the recovered catalyst........ S21-S22

Supplementary Fig. 22-32. ^{1}H and ^{13}C NMR spectra of monomer **2** S23-S28

Supplementary Fig.33-36. ^{1}H NMR spectra of **4**, **6**, **8** and **10**,...................... S28-S30

Supplementary Fig. 37-47. HPLC curves for product **4**, **6**, **8**, and **10** S30-S35

Supplementary References.. S35-S36

Supplementary Methods

Measurements. NMR spectra were recorded using a Bruker 600 MHz or 400 MHz spectrometer {H} operated in the Fourier Transform mode. Chemical shifts are reported in delta (δ) units and expressed in parts per million (ppm) downfield from tetramethylsilane (TMS) using the residual solvent proton as an internal standard. Size exclusion chromatography (SEC) was performed on Waters 1515 pump and Waters 2414 differential refractive index (RI) detector (set at 40 °C) using a series of two linear TSK gel GMH_{HR}-H columns. Molecular weight (M_n) and its dispersity (M_w/M_n) data are reported relative to polystyrene standards. The eluent was tetrahydrofuran (THF) at a flow rate of 0.8 mL/min. FT-IR spectra were recorded on Perkin-Elmer Spectrum BX FT-IR system using KBr pellets. CD spectra were obtained in a 1.0 mm quartz cell at 25 °C using a JASCO J1500 spectropolarimeter. Absorption spectra were recorded on UNIC 4802 UV/vis double beam spectrophotometer in a 1.0 cm quartz cell at 25 °C. The optical rotations were measured in $CHCl_3$ at room temperature using a 10.0 cm quartz cell on a WZZ-2S polarimeter. Matrix assisted laser desorption ionizations with time of flight detection mass spectroscopy (MALDI-TOF MS) measurements were performed on a Bruker Reflex III using dithranol as a matrix and sodium trifluoroacetate as an ion source. High performance liquid chromatography (HPLC) with UV-vis detector was carried out on SHIMADZU LC-20AT equipment using chiral column.

Materials. All solvents were obtained from Sinopharm. Co. Ltd., and were purified by the standard procedures before use. In experiments that required dry solvents, tetrahydrofuran (THF), toluene, chloroform ($CHCl_3$) and dichloromethane (CH_2Cl_2) were dried using standard methods and distilled before use. All chemicals were purchased from Aladdin, Sinopharm, and Sigma-Aldrich Chemical Co. Ltd., and were used as received otherwise denoted. The 4-methoxy-phenylacetylene Pd(II) catalyst (alkyne-Pd(II)), monomer **1**, and the phenyl isocyanide bearing pentafluorophenol ester (**16**) were prepared following the reported procedures, and the structures were confirmed by ^{1}H NMR.[1-4] Monomer **2** was prepared according to Scheme S1.

Supplementary Discussion

Synthesis of monomer 2.

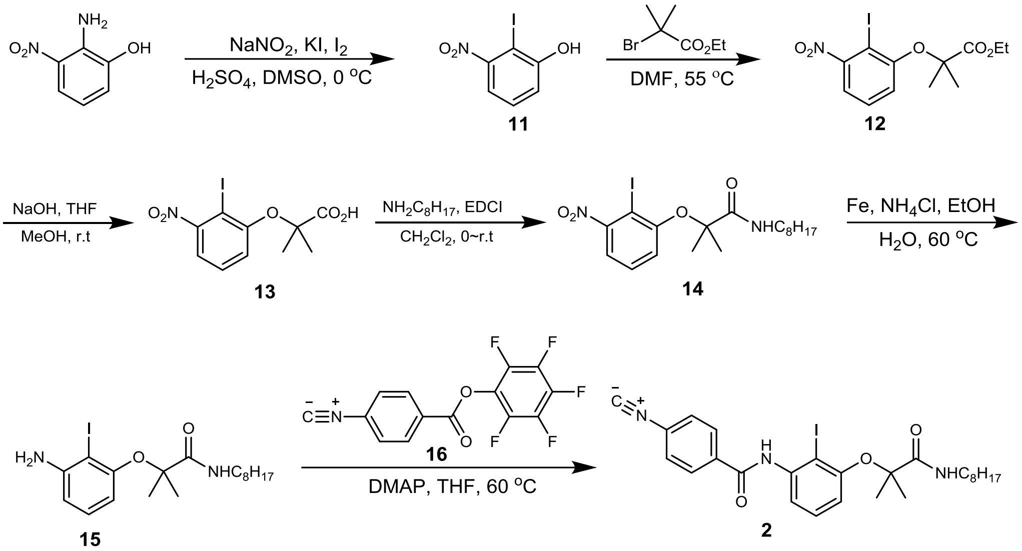

Supplementary Fig. 1 Synthetic Route for Monomer 2

Synthesis of ***11***. The compound was prepared following the reported literature with modification.[5] Into a solution of 2-amino-3-nitrophenol (3.02 g, 19.6 mmol) in dimethyl sulfoxide (DMSO, 50 mL) was added aq. 30% H_2SO_4 (100 mL). The mixtures were stirred for 1 h and cooled to 0 °C with an ice-water bath, the mixture was treated with a solution of $NaNO_2$ (1.99 g, 28.7 mmol) in deionized H_2O (10 mL) for 15 minutes. The reaction mixture was stirred at 0 °C for 1 h, after which a solution of KI (4.78 g, 28.8 mmol) and I_2 (3.65 g, 14.4 mmol) in deionized H_2O (30 mL) was added. After stirring at room temperature for 1 h, another batch of KI (1.60 g, 9.60 mmol) and I_2 (1.83 g, 7.20 mmol) in deionized H_2O (30 mL) was then added. The reaction mixture was stirred at room temperature for another 1 h and 80 °C for 2 h. Subsequently, the mixture was poured into ethyl acetate (200 mL) and was washed sequentially with brine, 10% $NaHSO_3$, and water. The organic phase was dried over Na_2SO_4, filtered, and concentrated. The residue was purified by flash chromatography on silica gel (petroleum ether/ethyl acetate, v/v = 6/1) to give **11** as a brown yellow solid in 61% yield. ^1H NMR (600 MHz, $CDCl_3$, 25 °C): δ 7.44 (d, J = 8.0 Hz, 1H), 7.37 (t, J = 8.0 Hz, 1H), 7.24 (d, J = 8.2 Hz, 1H), 6.00 (s, 1H).

Synthesis of ***12***. A mixture of **11** (3.05 g, 11.32 mmol), 2-bromoisobutyric acid ethyl

ester (2.64 g, 13.58 mol) and K_2CO_3 (7.81 g, 56.60 mol) in DMF (30 mL) was stirred at 80 °C for 24 h. After cooled to room temperature, the mixture was poured into ethyl acetate (50 mL) and washed with water. The organic phase was dried over Na_2SO_4, filtered, and concentrated. The residue was purified by flash chromatography on silica gel (petroleum ether/ethyl acetate, v/v = 8/1) to give **12** as a yellow oil in 89% yield. ^{1}H NMR (600 MHz, $CDCl_3$, 25 °C): δ 7.34–7.29 (m, 2H), 6.88 (dd, J = 6.4, 3.2 Hz, 1H), 4.25 (q, J = 7.2 Hz, 2H), 1.69 (s, 6H),1.25 (t, J = 7.2 Hz, 3H); ^{13}C NMR (150 MHz, $CDCl_3$, 25 °C): δ 173.33, 157.33, 156.78, 155.85, 129.30, 119.33, 117.94, 84.44, 81.83, 62.26, 25.29, 14.07; FT-IR (KBr, cm^{-1}): ν 2996, 2973, 2953, 2887, 1723, 1676, 1582, 1523, 1448, 1385, 1354, 1267, 1169, 1134, 993, 792, 761, 737, 647, 619, 541; MS *m/z* calcd for $C_{12}H_{13}INO_5$ ([M – H]$^-$): 377.99; Found: 377.98; Anal. Calcd (%) for $C_{12}H_{14}INO_5$: C, 38.01; H, 3.72; N, 3.69. Found (%): C, 38.05; H, 3.74; N, 3.69.

Synthesis of ***13***. To a solution of **12** (4.48 g, 11.81 mmol) in THF (30 mL) and MeOH (30 mL) was added aq. NaOH (2N, 30 mL) and stirred overnight at room temperature. The reaction mixture was cooled to 0 °C, quenched with 1N HCl and extracted with ethyl acetate. The organic layers were dried over anhydrous $MgSO_4$ and the solvents were removed in *vacuo* to give **13** as a yellow solid in 92% yield. ^{1}H NMR (600 MHz, $CDCl_3$, 25 °C): δ 7.37 (d, J = 9.2 Hz, 2H), 7.06 (q, J = 8.6 Hz, 1H), 1.75 (s, 6H); ^{13}C NMR (150 MHz, $CDCl_3$, 25 °C): δ 178.68, 156.42, 155.84, 129.50, 120.31, 118.62, 85.05, 81.66, 25.22; FT-IR (KBr, cm^{-1}): ν 3087, 2987, 2558, 1701, 1574, 1519, 1446, 1342, 1286, 1257, 1184, 1146, 998, 927, 872, 804, 783, 731, 603, 568; MS *m/z* calcd for $C_{10}H_9INO_5$ ([M – H]$^-$): 349.96; Found: 349.94; Anal. Calcd (%) for $C_{10}H_{10}INO_5$: C, 34.21; H, 2.87; N, 3.99. Found (%): C, 34.22; H, 2.87; N, 3.97.

Synthesis of ***14***. 1-(3-Dimethylaminopropyl)-3-ethylcarbodiimide hydrochloride (EDCI, 0.89 g, 4.68 mmol) were added to a solution of **13** (3.17 g, 8.85 mmol) in dry CH_2Cl_2 (50 mL). After the reaction mixture was stirred at 0 °C for 30 min under N_2, octylamine (1.37 g, 10.62 mmol) was added to the mixture. The solution was stirred at room temperature for 8 h. Then the mixture was washed sequentially with H_2O, saturated aq. $NaHCO_3$, and brine. The organic phase was dried over Na_2SO_4, filtered,

and concentrated. The residue was purified by flash chromatography on silica gel (petroleum ether/ethyl acetate, v/v = 4/1) to give **14** as a yellow oil in 78% yield. ^{1}H NMR (600 MHz, $CDCl_3$, 25 °C): δ 7.41–7.32 (m, 2H), 7.06 (dd, J = 6.8, 2.8 Hz, 1H), 6.76 (t, J = 6.0 Hz, 1H), 3.38–3.26 (m, 2H), 1.65 (s, 6H), 1.56–1.50 (m, 2H), 1.30–1.20 (m, 10H), 0.87 (t, J = 7.0 Hz, 3H); ^{13}C NMR (150 MHz, $CDCl_3$, 25 °C): δ 174.36, 154.98, 148.70, 129.09, 109.29, 107.87, 83.47, 82.00, 39.45, 31.78, 29.50, 29.23, 29.20, 26.97, 25.13, 22.63, 14.10; FT-IR (KBr, cm^{-1}): ν 3410, 3314, 2951, 2855, 1649, 1528, 1447, 1361, 1265, 1190, 1140, 1031, 988, 826, 791, 730, 595; MS *m/z* calcd for $C_{18}H_{26}IN_2O_4$ ([M – H]$^-$): 461.10; Found: 461.15; Anal. Calcd (%) for $C_{18}H_{27}IN_2O_4$: C, 46.76; H, 5.89; N, 6.06. Found (%): C, 46.77; H, 5.92; N, 6.08.

Synthesis of ***15***. To a solution of **14** (2.50 g, 5.03 mmol) in EtOH (120 mL) and NH_4Cl (0.67 g, 12.57 mmol) in H_2O (40 mL) was added Fe powder (1.38 g, 25.15 mmol). The resulting suspension was stirred at 60 °C for 4 h. The reaction mixture was filtered and the filtrate was condensed to give **15** as a colorless oil in 58% yield. ^{1}H NMR (600 MHz, $CDCl_3$, 25 °C): δ 7.03–6.90 (m, 2H), 6.47 (d, J = 9.0 Hz, 1H), 6.31 (d, J = 8.0 Hz, 1H), 4.16 (s, 2H), 3.32 (q, J = 3.2 Hz, 2H), 1.61 (s, 6H), 1.56–1.48 (m, 2H), 1.32–1.25 (m, 10H), 0.87 (t, J = 7.0 Hz, 3H); ^{13}C NMR (150 MHz, $CDCl_3$, 25 °C): δ 174.36, 154.98, 148.70, 129.09, 109.29, 108.74, 83.47, 82.00, 39.45, 31.78, 29.50, 29.20, 26.97, 25.13, 22.63, 14.10; FT-IR (KBr, cm^{-1}): ν 3450, 3390, 3319, 3193, 2951, 2905, 2845, 1649, 1625, 1591, 1518, 1464, 1440, 1370, 1250, 1156, 1021, 1008, 758, 716, 583; MS *m/z* calcd for $C_{18}H_{28}IN_2O_2$ ([M – H]$^-$): 431.13; Found: 431.15; Anal. Calcd (%) for $C_{18}H_{29}IN_2O_2$: C, 50.01; H, 6.76; N, 6.48. Found (%): C, 50.00; H, 6.76; N, 6.51.

Synthesis of ***2***. Phenyl isocyanide bearing pentafluorophenol ester (**16**) was prepared according to the reported procedure and the structure was confirmed by ^{1}H NMR.[2] A round bottom flask was charged with **16** (0.78 g, 2.49 mmol), **15** (1.15 g, 2.49 mmol), dimethylaminopyridine (DMAP, 0.40 g, 3.23 mmol), and anhydrous THF (20 mL). The resulting mixture was stirred at 60 °C for 24 h. Then the solvent was removed by evaporation under reduced pressure. The residue was dissolved in CH_2Cl_2 (30 mL) and washed successively with H_2O, saturated aq. $NaHCO_3$, and brine. After dried over

anhydrous Na_2SO_4 and filtration, the solvent was removed by evaporation under reduced pressure. The crude product was purified by flash chromatography on silica gel (petroleum ether/ethyl acetate, v/v = 4/1) to give **2** as a white solid in 35% yield. ^{1}H NMR (600 MHz, $CDCl_3$, 25 °C): δ 8.42 (s, 1H), 8.15 (d, J = 9.5 Hz, 1H), 8.02 (d, J = 8.5 Hz, 2H), 7.59–7.51 (m, 2H), 7.30 (t, J = 8.2 Hz, 1H), 6.81–6.68 (m, 2H), 3.33 (q, J = 7.0 Hz, 2H), 1.63 (s, 6H), 1.56–1.51 (m, 2H), 1.29–1.25 (m, 10H), 0.87 (t, J = 7.0 Hz, 3H); ^{13}C NMR (150 MHz, $CDCl_3$, 25 °C): δ 173.84, 168.01, 164.46, 155.17, 140.90, 135.28, 130.43, 128.52, 126.64, 116.20, 113.97, 89.64, 83.96, 39.52, 31.77, 29.51, 29.21, 29.20, 26.95, 25.24, 22.62, 14.10; FT-IR (KBr, cm^{-1}): ν 3302, 2956, 2916, 2850, 2113, 1680, 1646, 1569, 1520, 1491, 1458, 1417, 1280, 1262, 1142, 1024, 849, 770, 758, 638, 568, 556; MS *m/z* calcd for $C_{26}H_{31}IN_3O_3$ ([M – H]$^-$): 560.14; Found: 560.15; Anal. Calcd (%) for $C_{26}H_{32}IN_3O_3$: C, 55.62; H, 5.74; N, 7.48. Found (%): C, 55.66; H, 5.76; N, 7.49.

Polymerization procedure:

Synthesis of M-poly-L-1$_{150}$ and P-poly-L-1$_{50}$. These polymers were prepared according to the reported procedure.[3,4] Monomer L-**1** (600 mg, 1.68 mmol) and alkyne-Pd(II)-catalyst (8.55 mg, 0.0168 mmol) were dissolved in dry $CHCl_3$ (7.5 mL). The solution was stirred for 8 h at 55 °C, then cooled to ambient temperature and precipitated by methanol. The precipitated polymer was collected and dried in vacuum. The afforded crude polymer was suspended in acetone (180 mL) for 3 h with stirring and was filtrated. The filtrate was dried by evaporation under reduced pressure, afforded the expected *P*-poly-L-**1**$_{50}$ (52 mg, 11% yield). SEC: M_n = 20.3 kDa, M_w/M_n = 1.14; ^{1}H NMR (600 MHz, $CDCl_3$, 25 °C): δ 9.34–7.89 (br, NH), 7.10–5.47 (br, ArH), 5.05–3.65 (br, CH and CH_2), 2.12–0.61 (br, CH_2 and CH_3); FT-IR (KBr, cm^{-1}): ν 2919 ($\nu_{C–H}$), 2849 ($\nu_{C–H}$), 1744 ($\nu_{NHC=O}$), 1633 ($\nu_{OC=O}$), 1604 ($\nu_{C=N}$); $[\alpha]^{25}_D$ = 1788 (0.1, $CHCl_3$). The filter cake was dissolved in $CHCl_3$ (6 mL) and then precipitated in acetone. The precipitate solid was collected by filtration and dried in vacuum. After this procedure was repeated 3 times, the left-handed *M*-poly-L-**1**$_{150}$ was obtained (339 mg, 60%). SEC: M_n = 57.7 kDa, M_w/M_n = 1.10; ^{1}H NMR (600 MHz, $CDCl_3$, 25 °C): δ 9.24–8.00 (br,

NH), 7.16–5.70 (br, ArH), 4.88–3.50 (br, CH and CH_2), 1.95–0.45 (br, CH_2 and CH_3); FT-IR (KBr, cm^{-1}): ν 2925 ($\nu_{C–H}$), 2857 ($\nu_{C–H}$), 1751 ($\nu_{NHC=O}$), 1636 ($\nu_{OC=O}$), 1600 ($\nu_{C=N}$); $[\alpha]^{25}_{D}$ = –1820 (0.1, $CHCl_3$).

Synthesis of M-poly(L-1$_{150}$-b-2$_m$) and P- poly(L-1$_{50}$-b-2$_m$). Taking *M*-poly(L-**1**$_{150}$-*b*-**2**$_{10}$) as an example, the solution of **2** (8.13 mg, 0.014 mmol) and *M*-poly-L-**1**$_{150}$ (50 mg) in $CHCl_3$ (1.0 mL) was stirred for 10 h at 55 °C, then cooled to 25 °C and precipitated in acetone. After centrifugation, the polymer was collected and dried in vacuum, which was re-dissolved in CH_2Cl_2 (1 mL) and cooled to 0 °C. To this solution, trifluoroacetic acid (22 μL, 0.3 mmol) was added. The mixture was warmed to 25 °C and stirred for 12 h. Then, the solution was precipitated into methanol, and the precipitated polymer was collected and dried to afford a yellow solid *M*-poly(L-**1**$_{150}$-*b*-**2**$_{10}$) (46 mg, 80% yield). SEC: M_n = 63.1 kDa, M_w/M_n = 1.15. ^{1}H NMR (600 MHz, $CDCl_3$, 25 °C): δ 8.95–8.15 (br, NH), 7.45–4.80 (br, ArH), 4.93–3.78 (br, CH and CH_2), 3.64–2.89 (br, CH_2), 2.02–0.65 (br, CH_2 and CH_3); FT-IR (KBr, cm^{-1}): ν 2921 ($\nu_{C–H}$), 2853 ($\nu_{C–H}$), 1750 ($\nu_{NHC=O}$), 1626 ($\nu_{OC=O}$), 1600 ($\nu_{C=N}$); $[\alpha]^{25}_{D}$ = –1906 (0.1, $CHCl_3$).

P-poly(L-**1**$_{50}$-*b*-**2**$_{10}$) was prepared followed the similar procedure in 82% yield by using *P*-poly-L-**1**$_{50}$ as macroinitiator. SEC: M_n = 25.3 kDa, M_w/M_n = 1.18; ^{1}H NMR (600 MHz, $CDCl_3$, 25 °C): δ 8.87–8.13 (br, NH), 7.67–5.35 (br, ArH), 5.02–3.75 (br, CH and CH_2), 3.58–2.95 (br, CH_2), 2.63–0.66 (br, CH_2 and CH_3); FT-IR (KBr, cm^{-1}): ν 2929 ($\nu_{C–H}$), 2852 ($\nu_{C–H}$), 1749 ($\nu_{NHC=O}$), 1630 ($\nu_{OC=O}$), 1609 ($\nu_{C=N}$); $[\alpha]^{25}_{D}$ = 1894 (0.1, $CHCl_3$).

Synthesis of poly-2$_{20}$. A 10 mL oven dried and nitrogen-filled flask was charged with monomer **2** (100 mg, 0.17 mmol), alkyne-Pd(II) catalyst (4.31 mg, 0.0085 mmol), dry $CHCl_3$ (1.0 mL), and a stir bar. The resulting mixture was stirred at 55 °C for 10 h and then cooled to room temperature. The solution was precipitated into methanol. The precipitated solid was collected by centrifugation and dried in vacuum at room temperature overnight, afforded poly-**2**$_{20}$ as a yellow solid (81 mg, 79% yield). SEC: M_n = 11.2 kDa, M_w/M_n = 1.17; ^{1}H NMR (600 MHz, $CDCl_3$, 25 °C): δ 8.37–5.45 (br,

NH and ArH), 3.84–2.90 (br, CH_2), 1.90–0.55 (br, CH_2 and CH_3); FT-IR (KBr, cm^{-1}): ν 2924 ($\nu_{C–H}$), 2854 ($\nu_{C–H}$), 1658 ($\nu_{NHC=O}$), 1579 ($\nu_{C=N}$).

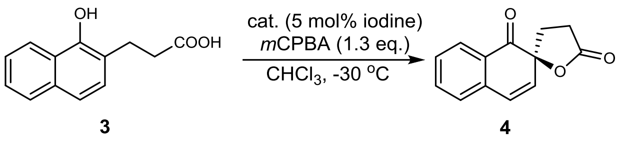

Supplementary Fig. 2 Asymmetric reaction of 3

Procedures for asymmetric catalysis:

Compound **3** was prepared according to the reported procedure and the structure was confirmed by ^{1}H NMR.[6] The compound **3** (4.28 mg, 0.02 mmol) was dissolved in $CHCl_3$ (2.0 mL) and cooled to 0 °C. Then *M*-poly(L-$\mathbf{1}_{150}$-*b*-$\mathbf{2}_{10}$) (5 mol% of the phenyl iodine pendants) and *meta*-chloroperoxybenzoic acid (*m*CPBA, 4.47 mg, 0.03 mmol) were added consecutively. The reaction mixture was stirred at –30 °C for 3 days. Saturated $Na_2S_2O_3$ and 1 M aq. Na_2CO_3 were added and the phases were separated. The aqueous layer was extracted with ethyl acetate and the combined organic layers were washed with brine and dried over Na_2SO_4. The solvent was removed under reduced pressure and the crude product was purified by column chromatography on silica gel (petroleum ether/ethyl acetate, v/v = 5/1) to afford compound **4** as a white solid. ^{1}H NMR (600 MHz, $CDCl_3$, 25 °C): δ 8.02 (d, J = 6.8 Hz, 1H), 7.63 (td, J = 7.6, 1.4 Hz, 1H), 7.41 (td, J = 7.6, 1.2 Hz, 1H), 7.26 (d, J = 12.0 Hz, 1H), 6.66 (d, J = 9.8 Hz, 1H), 6.21 (d, J = 9.8 Hz, 1H), 2.91 (ddd, J = 17.6, 11.2, 9.6 Hz, 1H), 2.60 (ddd, J = 17.6, 9.6, 2.2 Hz, 1H), 2.42 (ddd, J = 13.6, 9.6, 2.2 Hz, 1H), 2.21–2.16 (m, 1H).

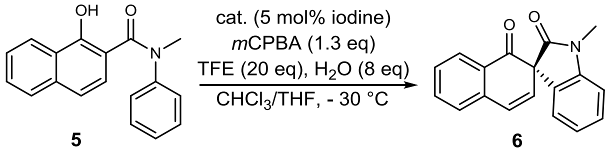

Supplementary Fig. 3 Asymmetric reaction of 5

Compound **5** was prepared according to the reported procedure and the structure was confirmed by ^{1}H NMR.[7] A 10 mL oven-dried and nitrogen-filled flask was charged with the *M*-poly(L-$\mathbf{1}_{150}$-*b*-$\mathbf{2}_{10}$) (5 mol% of the phenyl iodine pendants), *m*CPBA (4.47 mg, 0.03 mmol), 2,2,2-trifluoroethanol (TFE, 30 μL, 0.4 mmol), and H_2O (3 μL, 0.16 mmol)

was cooled to –30 °C. Then a solution of **5** (5.26 mg, 0.02 mmol) in $CHCl_3$ (1 mL) and THF (0.2 ml) was added. The reaction mixture was stirred at –30 °C for 6 days. Then the reaction was quenched by aqueous Na_2SO_3 (5 mL). The aqueous layer was extracted with CH_2Cl_2 (2 mL × 3). The combined organic layer was dried over Na_2SO_4 and the solvent was removed in *vacuo*. The residue was purified by flash column chromatography on silica gel (petroleum ether/ethyl acetate/CH_2Cl_2, 7/1/2, v/v/v) to afford **6** as a white solid. ^{1}H NMR (600 MHz, $CDCl_3$, 25 °C): δ 7.98 (d, J = 7.8 Hz, 1H), 7.64 (t, J = 6.8 Hz, 1H), 7.41–7.32 (m, 3H), 7.0–6.91 (m, 4H), 6.03 (d, J = 9.6 Hz, 1H), 3.29 (s, 3H).

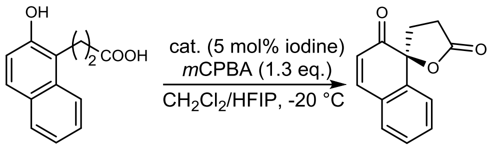

Supplementary Fig. 4 Asymmetric reaction of 7

Compound **7** was prepared according to the reported procedure and the structure was confirmed by ^{1}H NMR.[8] A 10 mL oven-dried and nitrogen-filled flask was charged with *M*-poly(L-$\mathbf{1}_{150}$-*b*-$\mathbf{2}_{10}$) (5 mol% of phenyl iodine pendants), *m*CPBA (4.47 mg, 0.03 mmol) and hexafluoroisopropanol (HFIP, 43 μL, 0.4 mmol) at –20 °C. Then a solution of **7** (5.26 mg, 0.02 mmol) in CH_2Cl_2 (2 mL) was added. After stirred at –20 °C for 5 days, the mixture was poured into aqueous solution of $Na_2S_2O_3$ and $NaHCO_3$ (5 mL). The aqueous layer was separated and extracted with CH_2Cl_2 (2 times). The combined organic layers were dried over anhydrous $MgSO_4$ and solvents were removed in *vacuo*. The residue was purified by flash column chromatography on silica gel (petroleum ether/ethyl acetate, v/v = 1/1) to afford **8** as a white solid. ^{1}H NMR (600 MHz, $CDCl_3$, 25 °C): δ 7.56 (d, J = 7.7 Hz, 1H), 7.47 (dd, J = 8.9, 5.2 Hz, 2H), 7.40 (t, J = 7.5 Hz, 1H), 7.35 (d, J = 7.6 Hz, 1H), 6.18 (d, J = 10.0 Hz, 1H), 2.92–2.79 (m, 1H), 2.74–2.56 (m, 2H), 2.15 (ddd, J = 12.5, 11.1, 9.6 Hz, 1H).

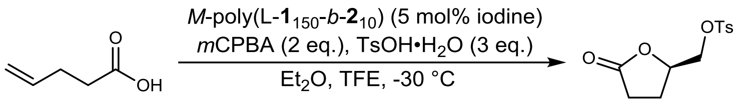

Supplementary Fig. 5 Asymmetric reaction of 9

The 4-pentenoic acid (**9**, 4.20 mg, 0.04 mmol) was dissolved in ethyl ether (Et_2O, 2 ml) in a flask. The *M*-poly(L-$\mathbf{1}_{150}$-*b*-$\mathbf{2}_{10}$) (5 mol% of the phenyl iodine pendants), *p*-toluenesulfonic acid monohydrate ($TsOH{\cdot}H_2O$, 22.80 mg, 0.12 mmol) and *m*CPBA (13.81 mg, 0.08 mmol) were then added. The solution was stirred for 6 days at –30 °C. The mixture was quenched with a saturated aqueous Na_2CO_3 solution and a saturated $Na_2S_2O_3$ aqueous solution. The aqueous layer was extracted 3 times with CH_2Cl_2. The combined organic layer was washed with brine, dried over $MgSO_4$, filtered and the solvent was removed *in vacuo*. The residue was purified by flash column chromatography on silica gel (petroleum ether/ethyl acetate, v/v = 1/1) to afford **10r** as a pale yellow oil.[9] ^{1}H NMR (600 MHz, $CDCl_3$, 25 °C): δ 7.78 (d, J = 6.2 Hz, 2H), 7.36 (d, J = 8.0 Hz, 2H), 4.71–4.67 (m, 1H), 4.18 (dd, J = 11.0, 3.2 Hz, 1H), 4.16–4.10 (m, 1H), 2.66–2.50 (m, 2H), 2.45 (s, 3H), 2.40–2.29 (m, 1H), 2.18–2.08 (m, 1H).

Supplementary Notes

Supplementary Table 1. Characterization Data for Helical Polyisocyanides[a]

run	catalyst	M_n[b] (kDa)	M_w/M_n[b]	Yield[c]	$\Delta\varepsilon_{364}$[d] (M^{-1} cm^{-1})	$[\alpha]^{25}_D$[e]
1	*M*-poly-L-**1**$_{150}$	57.7	1.10	60%	–21.02	–1820
2	*P*-poly-L-**1**$_{50}$	20.3	1.14	11%	20.62	1788
2	*M*-poly(L-**1**$_{150}$-*b*-**2**$_5$)	60.3	1.15	84%	–22.24	–1915
3	*M*-poly(L-**1**$_{150}$-*b*-**2**$_{10}$)	63.1	1.15	80%	–22.18	–1906
4	*M*-poly(L-**1**$_{150}$-*b*-**2**$_{15}$)	65.8	1.16	81%	–21.53	–1877
5	*M*-poly(L-**1**$_{150}$-*b*-**2**$_{20}$)	68.3	1.16	80%	–20.78	–1840
6	*P*-poly(L-**1**$_{50}$-*b*-**2**$_{10}$)	25.3	1.18	82%	21.87	1894
7	poly-**2**$_{20}$	11.2	1.17	79%	n.d.[f]	n.d.[f]

[a]These polymers were synthesized according to Scheme 1 in the main text. [b]The M_n and M_w/M_n were determined by SEC with equivalent to polystyrene standards. [c]Isolated yields. [d]The data were obtained from CD recorded in THF at 25 °C. [e]The optical rotations were measured in $CHCl_3$ (c = 0.1, 25 °C). [f]Not detected.

Supplementary Table 2. The Effect of Catalyst Loading on the Reaction[a]

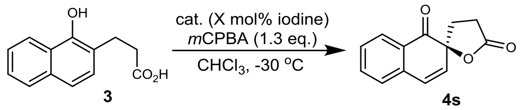

run	catalyst (eq.)	Yield[b]	*ee*[c]
1	0.1	77%	92%
2	1	78%	93%
3	5	81%	95%
4	10	81%	92%

[a]All reactions were carried carried out using **3** (0.02 mmol), *M*-poly(L-**1**$_{150}$-*b*-**2**$_{10}$) (X mol% of the phenyl iodine pendants), and *m*CPBA (0.03 mmol) in $CHCl_3$ (2.0 mL). [b]Yield of isolated products **4s**. [c]The *ee* values are referred to the major isomer determined by HPLC analysis using a chiral stationary phase.

Supplementary Table 3. Recycling *M*-poly(L-1$_{150}$-*b*-2$_{10}$) in Kita-spirolactonization of 3[a]

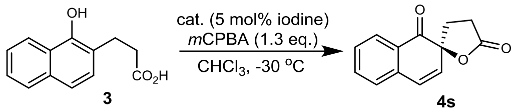

cycles	Recovered yield of catalyst[b]	Yield of **4**[c]	*ee*[d]
1	92%	85%	93%
2	89%	84%	94%
3	90%	83%	92%
4	88%	86%	95%
5	90%	85%	93%
6	89%	87%	94%
7	92%	82%	92%
8	91%	84%	92%
9	89%	86%	95%
10	87%	84%	92%

[a]All reactions were carried out using **3** (0.02 mmol), *M*-poly(L-**1**$_{150}$-*b*-**2**$_{10}$) (5 mol% of the phenyl iodine pendants), and *m*CPBA (0.03 mmol) in $CHCl_3$ (2.0 mL). [b]Yield of the recovered *M*-poly(L-**1**$_{150}$-*b*-**2**$_{10}$). [c]Yield of isolated product **4s**. [d]The *ee* values are referred to the major isomer determined by HPLC analysis using a chiral stationary phase.

Supplementary Table 4. Recycle Using *M*-poly(L-1$_{150}$-*b*-2$_{10}$) in the Spirocyclization of 5[a]

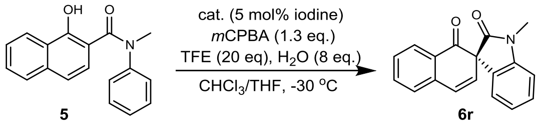

cycles	Recovered yield of catalyst[b]	yield of **6r**[c]	*ee*[d]
1	89%	62%	90%
2	90%	64%	89%
3	92%	61%	88%
4	88%	65%	89%
5	87%	63%	91%
6	88%	64%	91%
7	88%	64%	88%
8	91%	65%	90%
9	92%	63%	88%
10	88%	62%	89%

[a]All reactions were carried out using **5** (0.02 mmol), *M*-poly(L-**1**$_{150}$-*b*-**2**$_{10}$) (5 mol% of the phenyl iodine pendants), *m*CPBA (0.03 mmol), TFE (0.4 mmol), and H_2O (0.16 mmol) in $CHCl_3$ (1 mL) and THF (0.2 ml). [b]Yield of the recovered *M*-poly(L-**1**$_{150}$-*b*-**2**$_{10}$) catalyst. [c]Yield of isolated product **6r**. [d]The *ee* values are referred to the major isomer determined by HPLC analysis using a chiral stationary phase.

Supplementary Table 5. Yield and *ee* values of the asymmetric reactions of 3, 5, and 7 catalyzed by recycled *M*-poly(L-$\mathbf{1}_{150}$-*b*-$\mathbf{2}_{10}$)

cycle	yield[d] (%)	yield[e] (%)	*ee*[f] (%)
1[a]	92	85	93
2[b]	91	61	90
3[c]	89	82	85
4[a]	87	84	92
5[b]	90	63	89
6[c]	89	81	84

[a]The reactions were carried out using **3** (0.02 mmol), *M*-poly(L-$\mathbf{1}_{150}$-*b*-$\mathbf{2}_{10}$) (5 mol% of the phenyl iodine pendants), and *m*CPBA (0.03 mmol) in $CHCl_3$ (2.0 mL) at –30 °C. [b]The reactions were carried out using **5** (0.02 mmol), *M*-poly(L-$\mathbf{1}_{150}$-*b*-$\mathbf{2}_{10}$) (5 mol% of the phenyl iodine pendants), *m*CPBA(0.03 mmol), TFE (0.4 mmol), and H_2O (0.16 mmol) in $CHCl_3$ (1 mL) and THF (0.2 ml) at –30 °C. [c]The reactions were carried carried out using **7** (0.02 mmol), *M*-poly(L-$\mathbf{1}_{150}$-*b*-$\mathbf{2}_{10}$) (5 mol% of the phenyl iodine pendants), *m*CPBA (0.03 mmol), and HFIP (0.4 mmol) in CH_2Cl_2 (2.0 mL) at –20 °C. [d]Yield of the recovered *M*-poly(L-$\mathbf{1}_{150}$-*b*-$\mathbf{2}_{10}$) catalyst. [e]Yield of isolated products of the asymmetric reactions. [f]The *ee* values are referred to the major isomer determined by HPLC analysis using a chiral stationary phase.

Supplementary Table 6. Optimization of Sulfonyl-oxylactonization of 9[a]

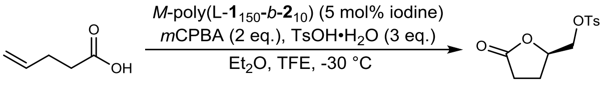

9 10

run	Sol.	Temp.	yield[b] (%)	*ee*[c] (%)
1	CH_2Cl_2	0 °C	78	36
2	$CHCl_3$	0 °C	75	37
3	Et_2O	0 °C	64	51
4	THF	0 °C	49	35
5	CH_3CN	0 °C	72	22
6[d]	Et_2O	0 °C	67	58
7[d]	Et_2O	-30 °C	72	74

[a]Unless otherwise denoted, all reactions were carried out **9** (0.04 mmol), *M*-poly(L-$\mathbf{1}_{100}$-*b*-$\mathbf{2}_{10}$) (5 mol %), TsOH·H_2O (0.12 mmol) and *m*CPBA (0.08 mmol) in specific solvent (2.0 mL). [b]Yield of isolated products. [c]The *ee* values are referred to the major isomer determined by HPLC analysis using a chiral stationary phase. [d]Using 20 equiv. of 2,2,2-Trifluoroethanol (TFE) as additive.

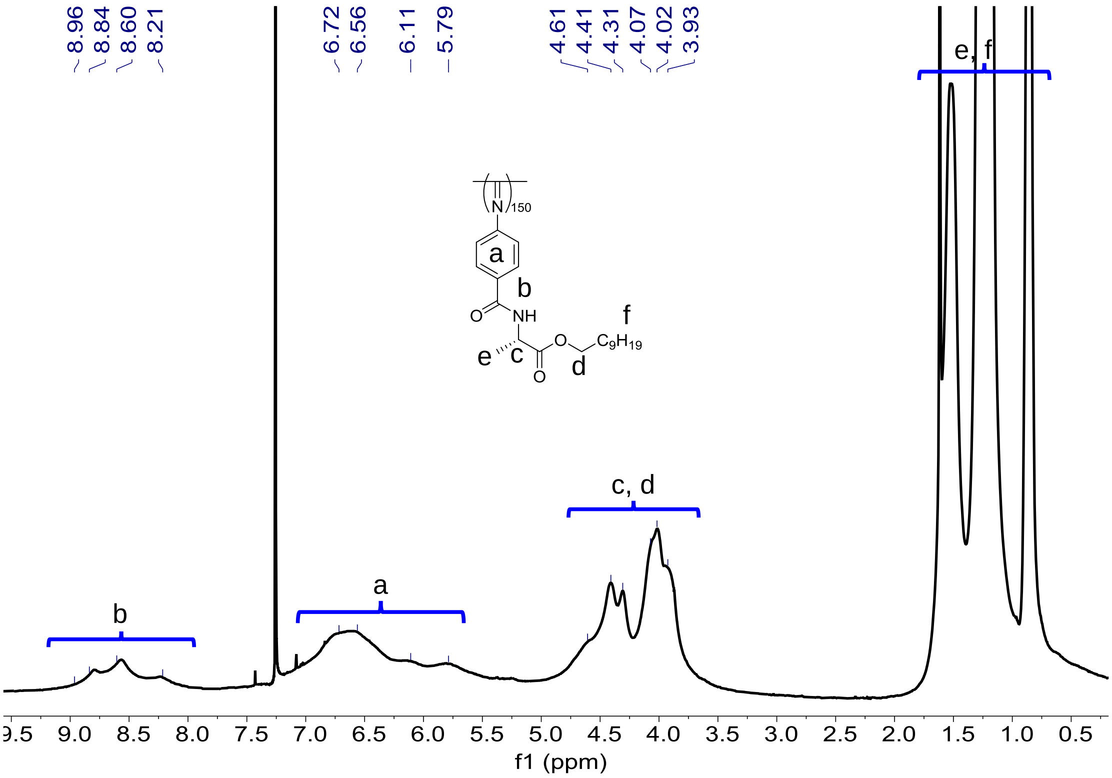

Supplementary Fig. 6 ^{1}H NMR (600 MHz) spectrum of *M*-poly-L-**1**$_{150}$ measured in $CDCl_3$ at 25 °C.

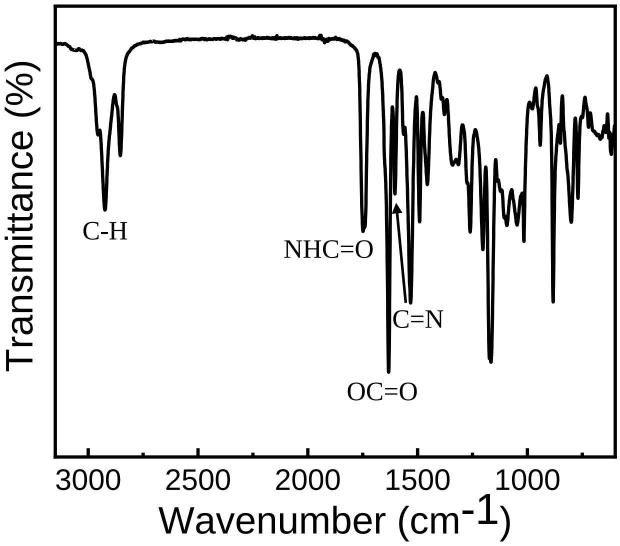

Supplementary Fig. 7 FT-IR spectrum of *M*-poly-L-**1**$_{150}$ measured at 25 °C using KBr pellets.

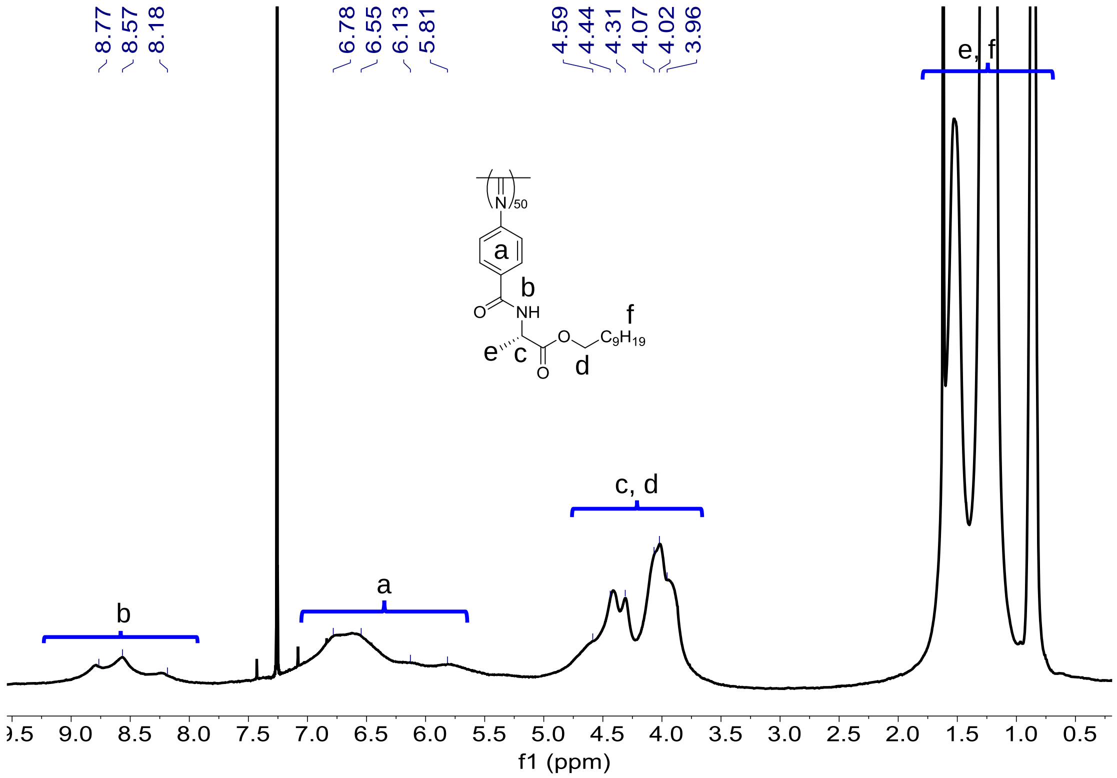

Supplementary Fig. 8 ^{1}H NMR (600 MHz) spectrum of *P*-poly-L-**1**$_{50}$ measured in $CDCl_3$ at 25 °C.

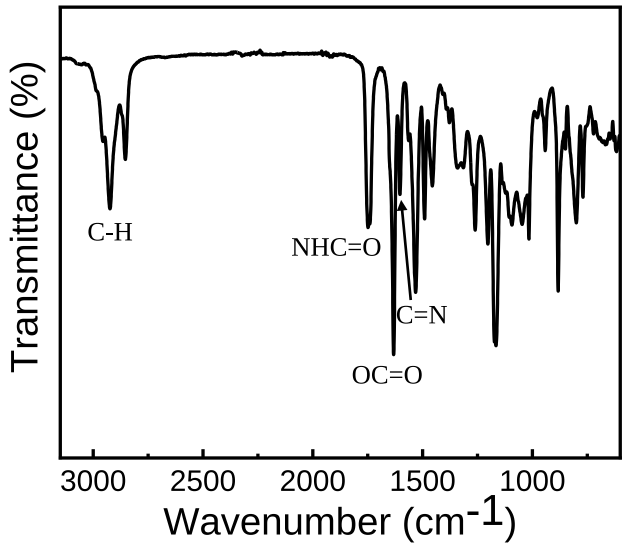

Supplementary Fig. 9 FT-IR spectrum of *P*-L-poly-**1**$_{50}$ measured at 25 °C using KBr pellets.

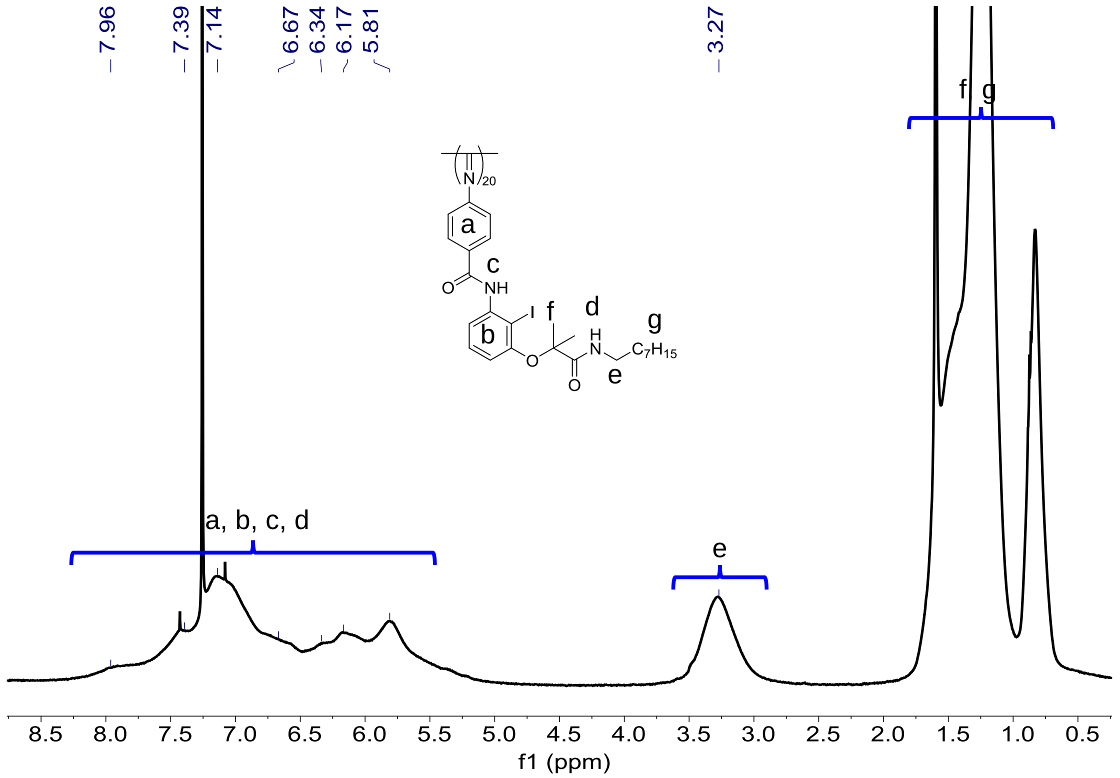

Supplementary Fig. 10 ^{1}H NMR (600 MHz) spectrum of poly-**2**$_{20}$ measured in $CDCl_3$ at 25 °C.

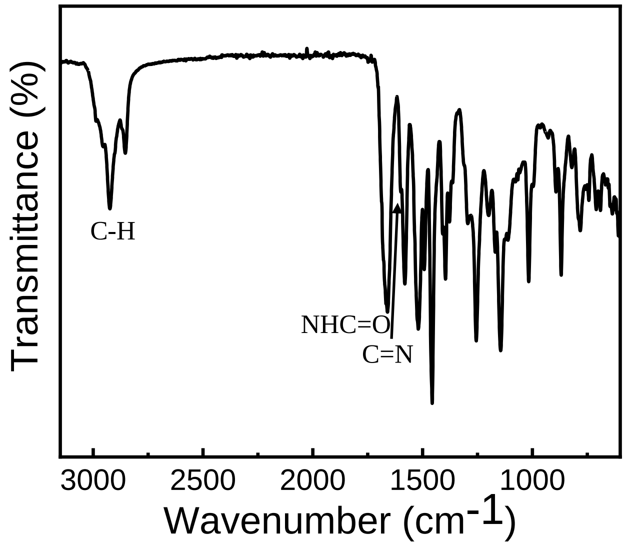

Supplementary Fig. 11 FT-IR spectrum of poly-**2**$_{20}$ measured at 25 °C using KBr pellets.

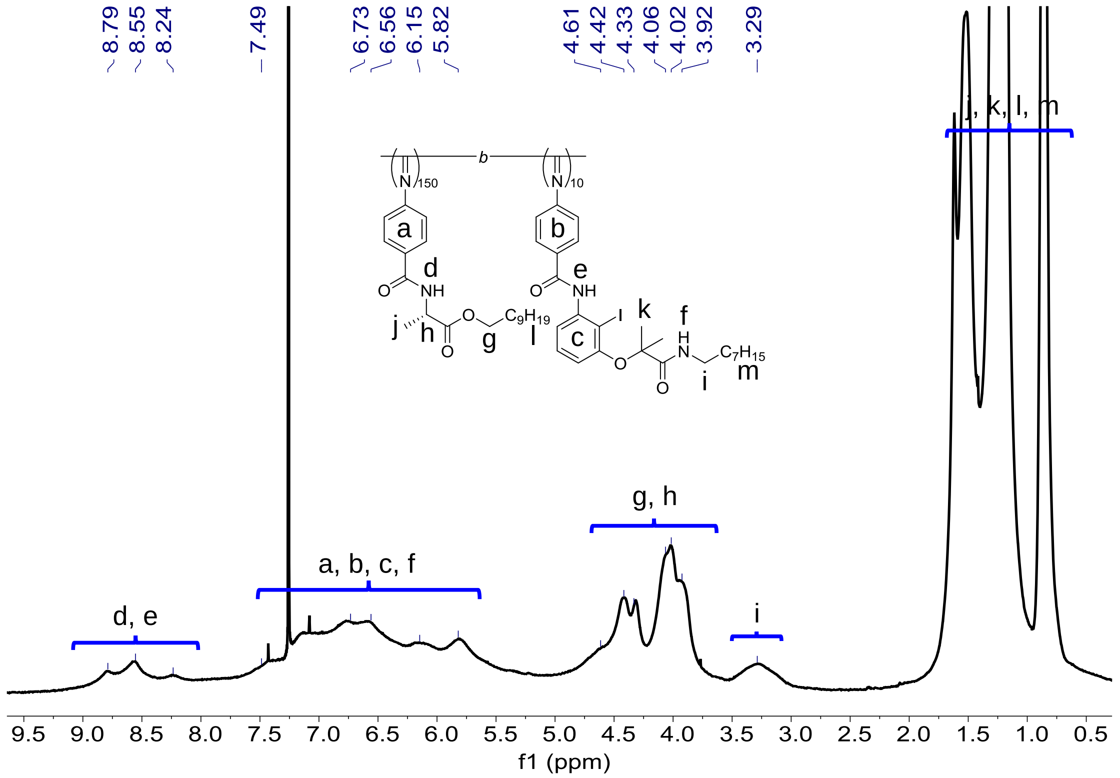

Supplementary Fig. 12 ^{1}H NMR (600 MHz) spectrum of *M*-poly(L-**1**$_{150}$-*b*-**2**$_{10}$) measured in $CDCl_3$ at 25 °C.

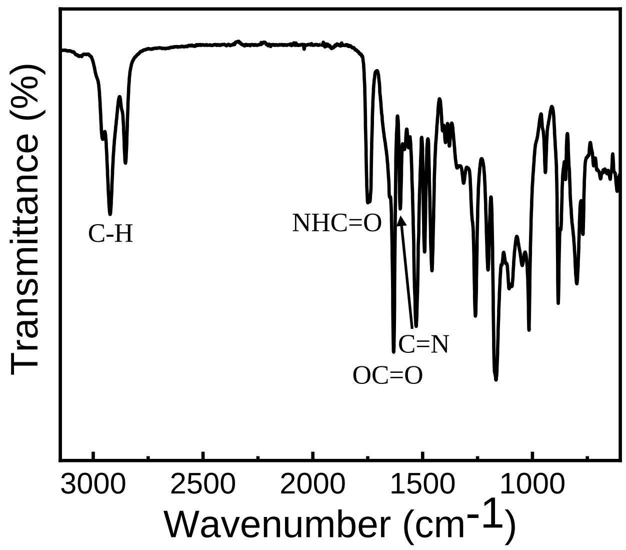

Supplementary Fig. 13 FT-IR spectrum of *M*-poly(L-**1**$_{150}$-*b*-**2**$_{10}$) measured at 25 °C using KBr pellets.

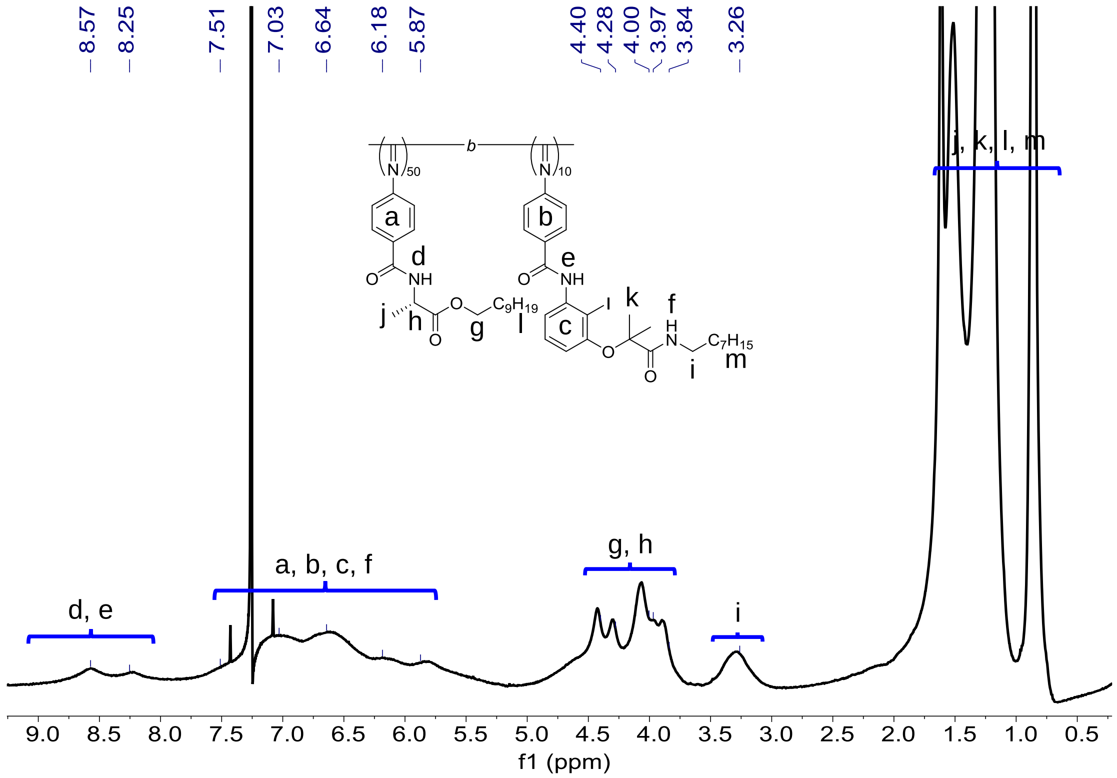

Supplementary Fig. 14 ^{1}H NMR (600 MHz) spectrum of *P*-poly(L-**1**$_{50}$-*b*-**2**$_{10}$) measured in $CDCl_3$ at 25 °C.

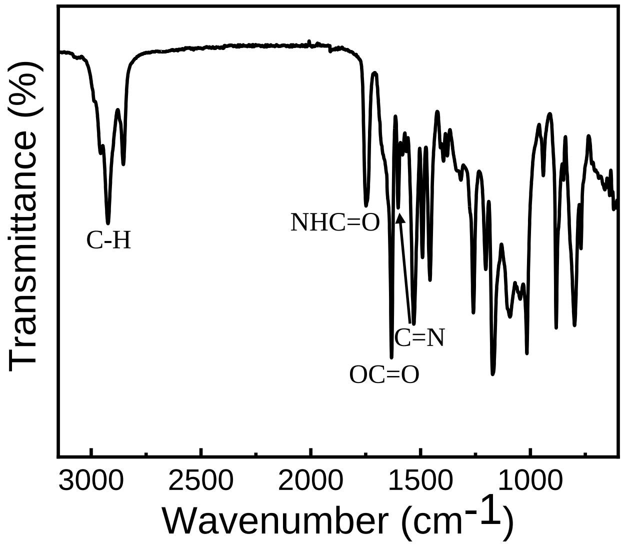

Supplementary Fig. 15 FT-IR spectrum of *P*-poly(L-**1**$_{50}$-*b*-**2**$_{10}$) measured at 25 °C using KBr pellets.

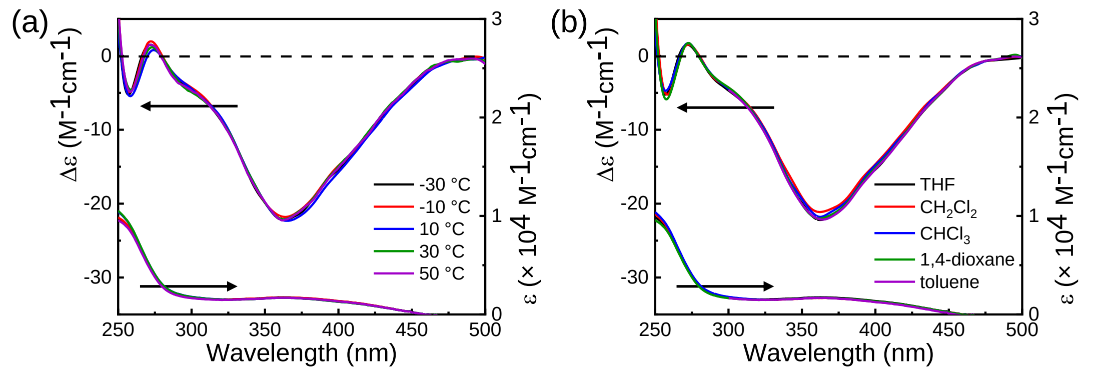

Supplementary Fig. 16 CD and UV-vis spectra of M-poly(L-**1**$_{150}$-*b*-**2**$_{10}$) measured in different solvents at room temperature (a), and in THF at different temperatures (b) (c = 0.2 mg/mL).

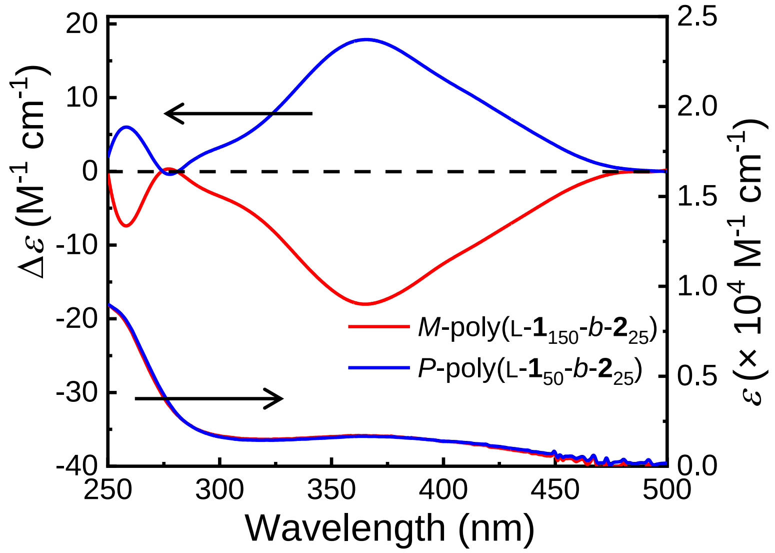

Supplementary Fig. 17 CD and UV-vis spectra of M-poly(L-**1**$_{150}$-*b*-**2**$_{25}$) and P-poly(L-**1**$_{50}$-*b*-**2**$_{25}$) in THF at 25 °C (0.20 mg/mL).

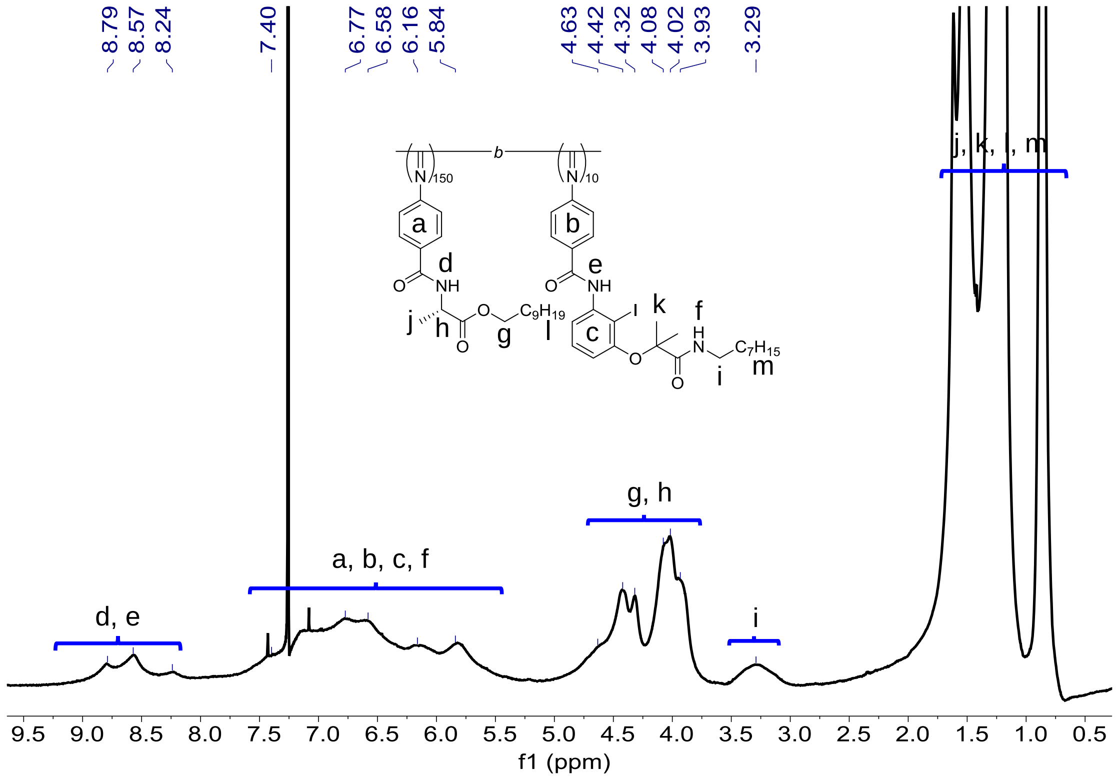

Supplementary Fig. 18 ^{1}H NMR (600 MHz) spectrum of the recovered *M*-poly(L-**1**$_{150}$-*b*-**2**$_{10}$) measured in $CDCl_3$ at 25 °C.

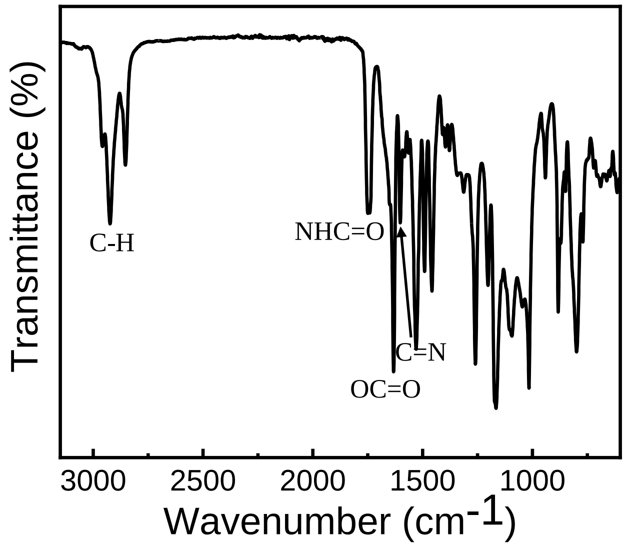

Supplementary Fig. 19 FT-IR spectrum of the recovered *M*-poly(L-**1**$_{150}$-*b*-**2**$_{10}$) measured at 25 °C using KBr pellets.

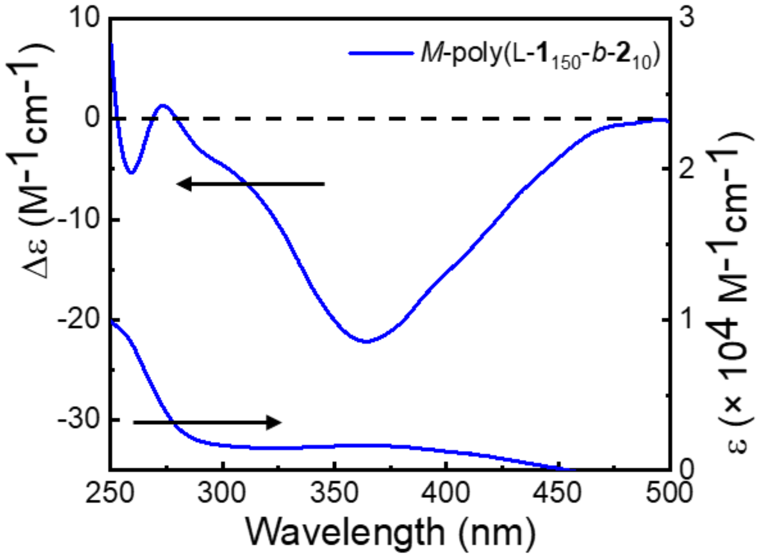

Supplementary Fig. 20 CD and UV-vis spectra of the recovered *M*-poly(L-$\mathbf{1}_{150}$-*b*-$\mathbf{2}_{10}$) (THF, 25 °C, 0.2 mg/mL).

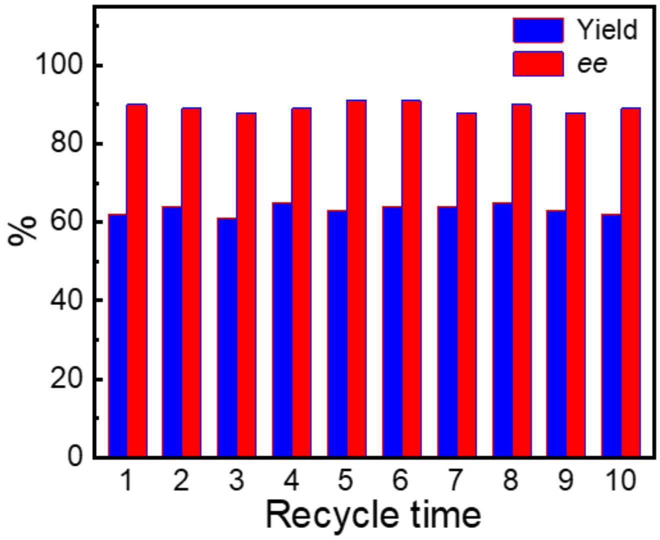

Supplementary Fig. 21 Yields and *ee* values of the dearomatizative spirocyclization of **5** catalyzed by recycled *M*-poly(L-$\mathbf{1}_{150}$-*b*-$\mathbf{2}_{10}$).

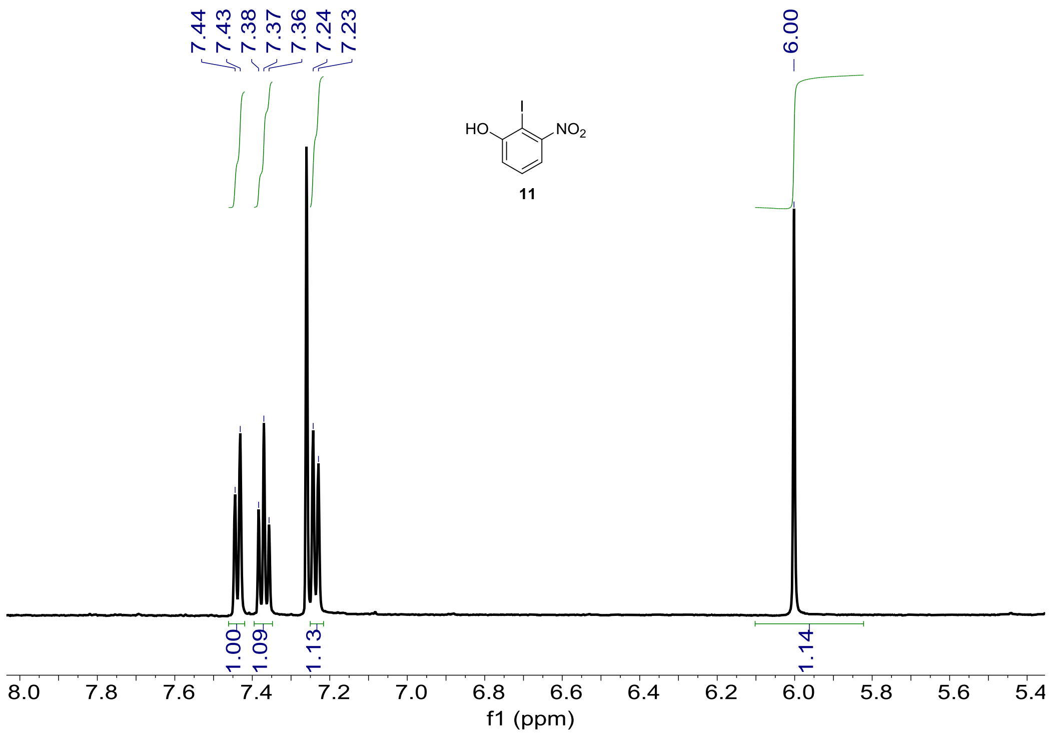

Supplementary Fig. 22 ^{1}H NMR (600 MHz) spectrum of **11** measured in $CDCl_3$ at 25 °C.

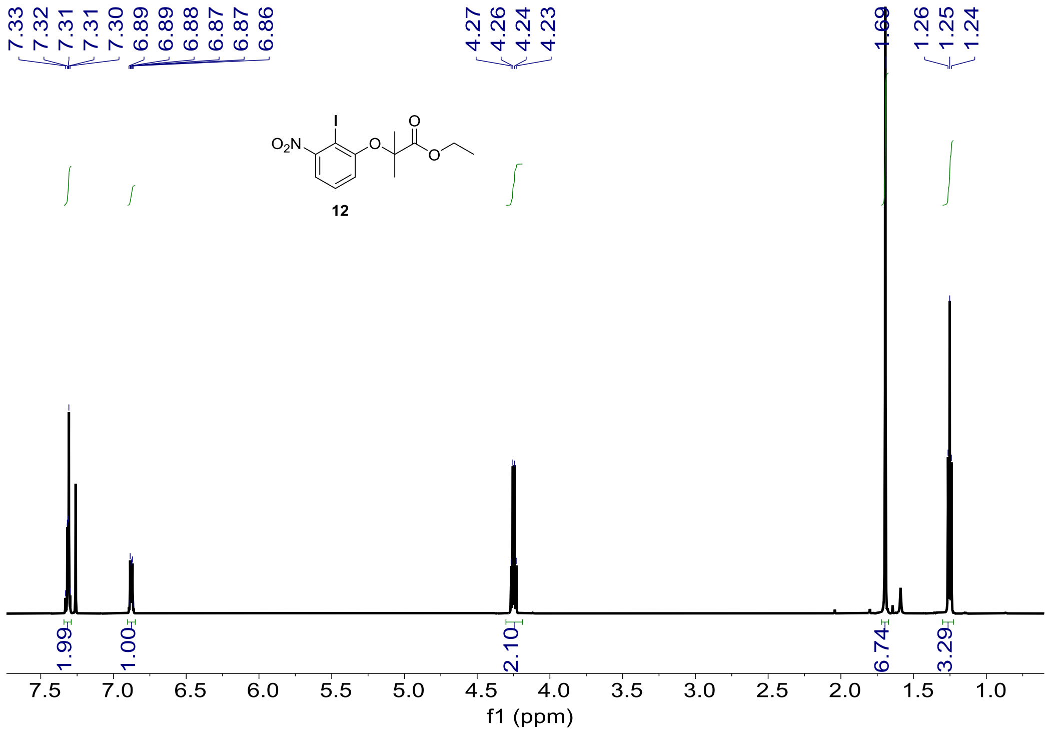

Supplementary Fig. 23 ^{1}H NMR (600 MHz) spectrum of **12** measured in $CDCl_3$ at 25 °C.

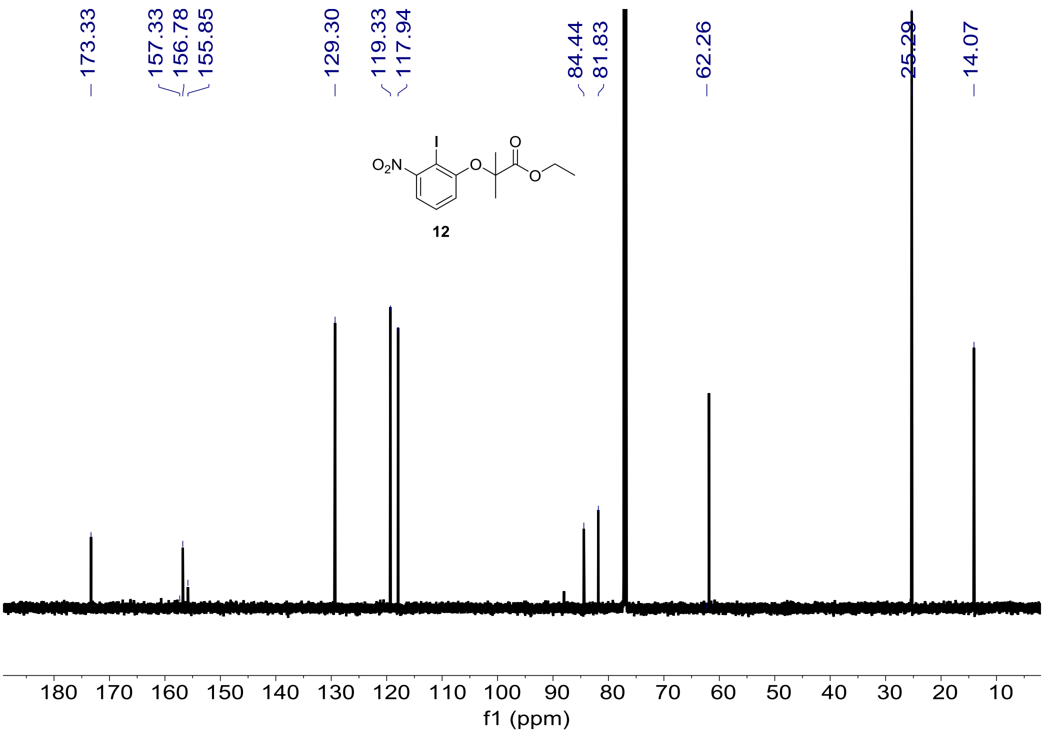

Supplementary Fig. 24 ^{13}C NMR (150 MHz) spectrum of **12** measured in $CDCl_3$ at 25 °C.

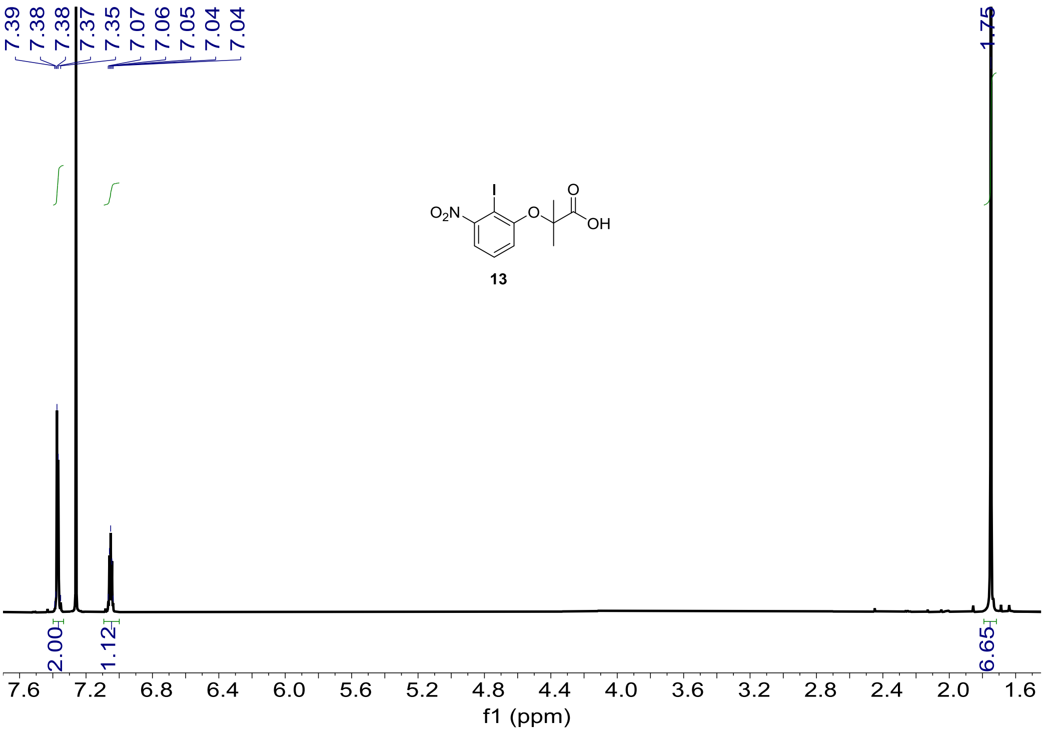

Supplementary Fig. 25 ^{1}H NMR (600 MHz) spectrum of **13** measured in $CDCl_3$ at 25 °C.

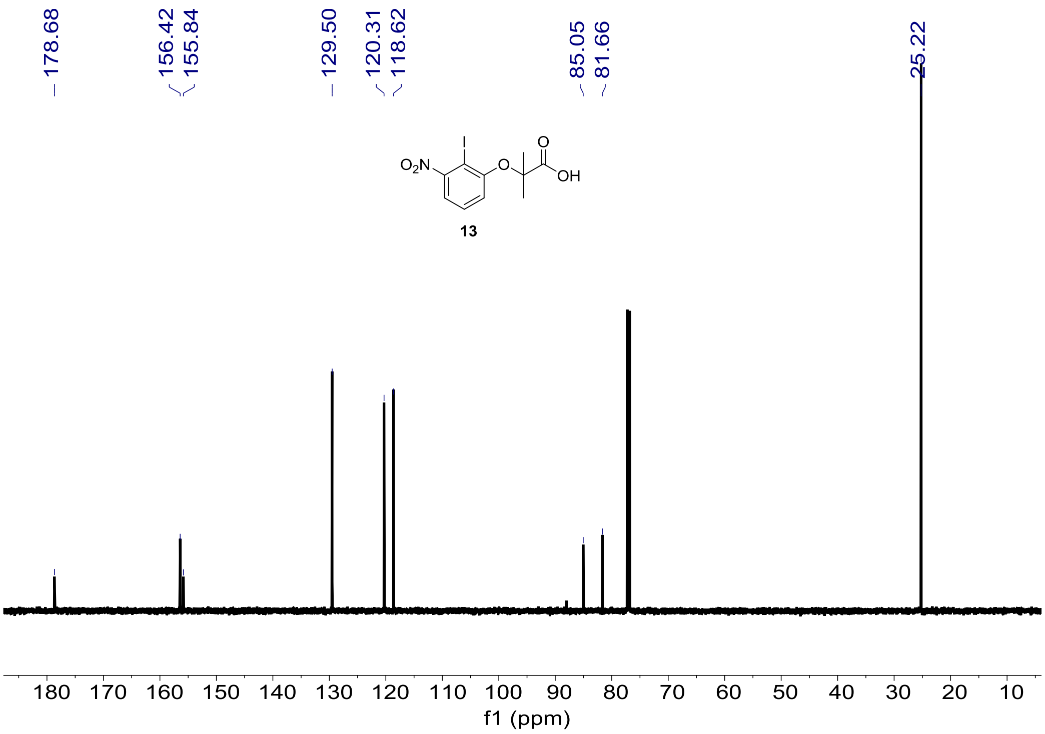

Supplementary Fig. 26 ^{13}C NMR (150 MHz) spectrum of **13** measured in $CDCl_3$ at 25 °C.

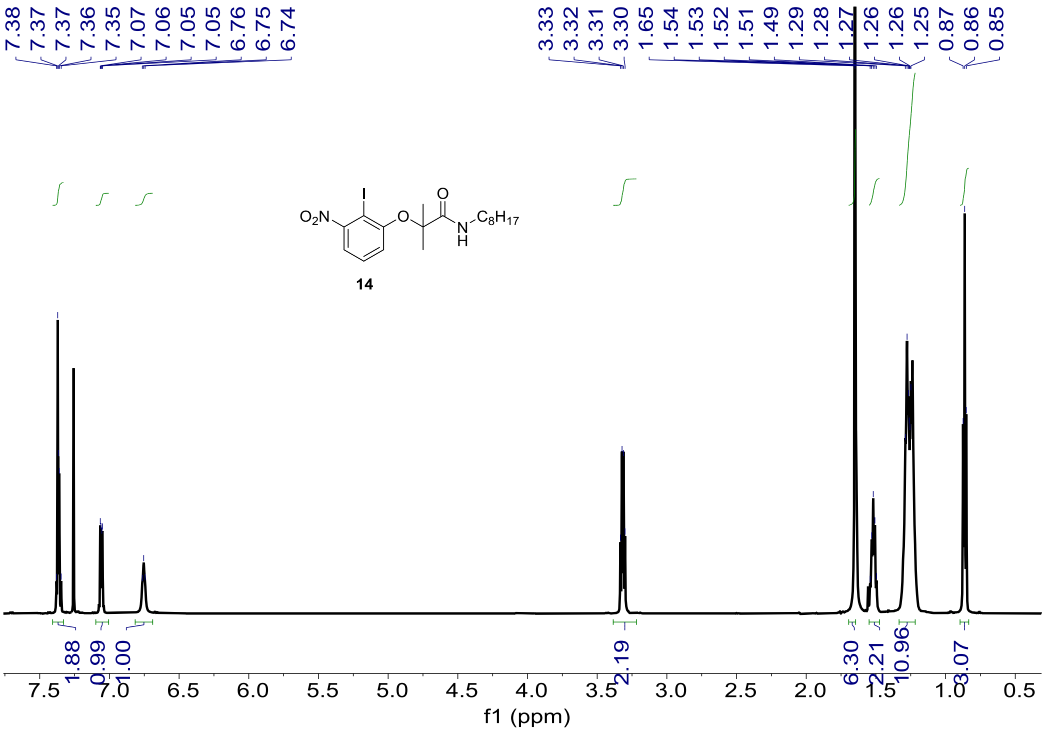

Supplementary Fig. 27 ^{1}H NMR (600 MHz) spectrum of **14** measured in $CDCl_3$ at 25 °C.

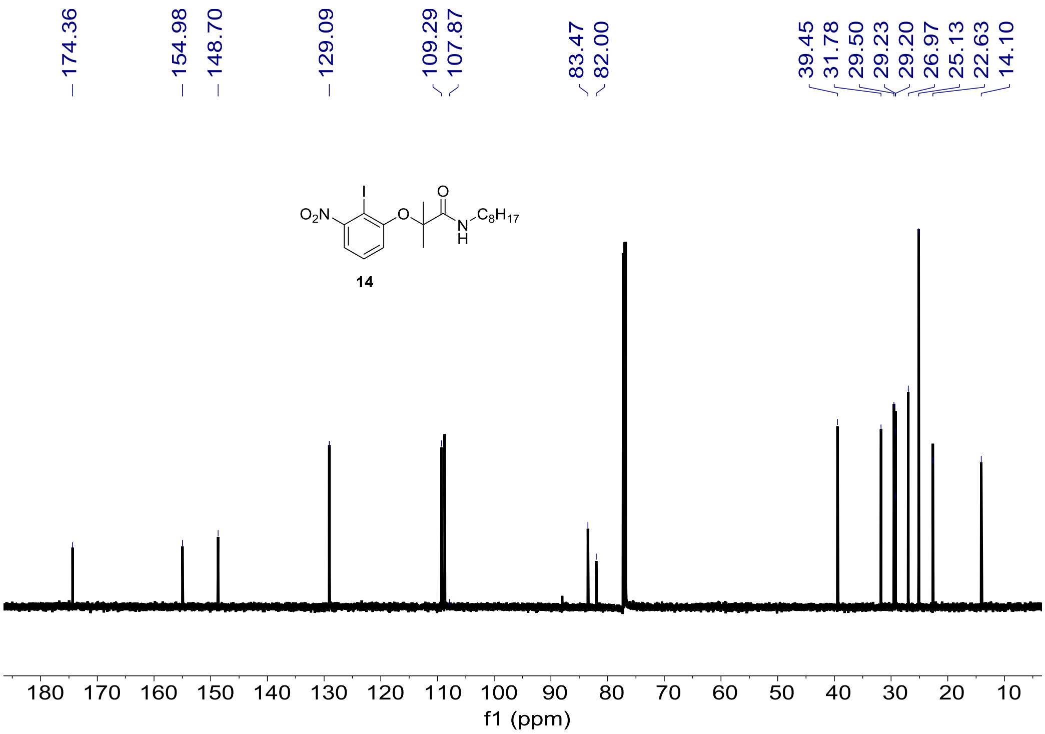

Supplementary Fig. 28 ^{13}C NMR (150 MHz) spectrum of **14** measured in $CDCl_3$ at 25 °C.

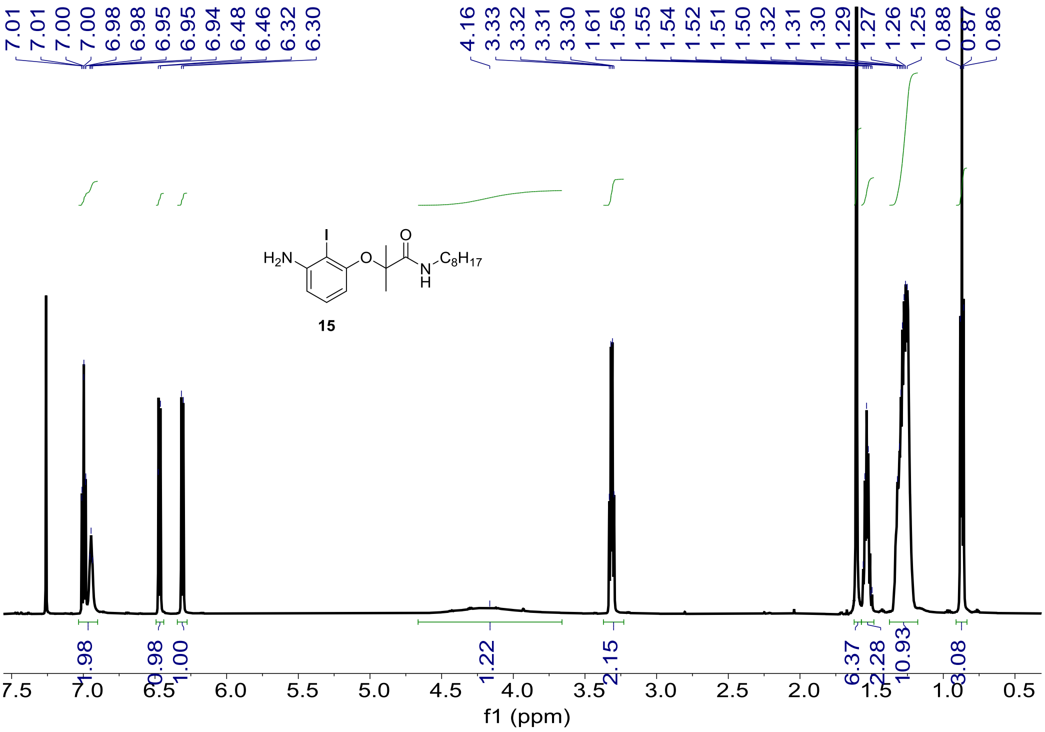

Supplementary Fig. 29 ^{1}H NMR (600 MHz) spectrum of **15** measured in $CDCl_3$ at 25 °C.

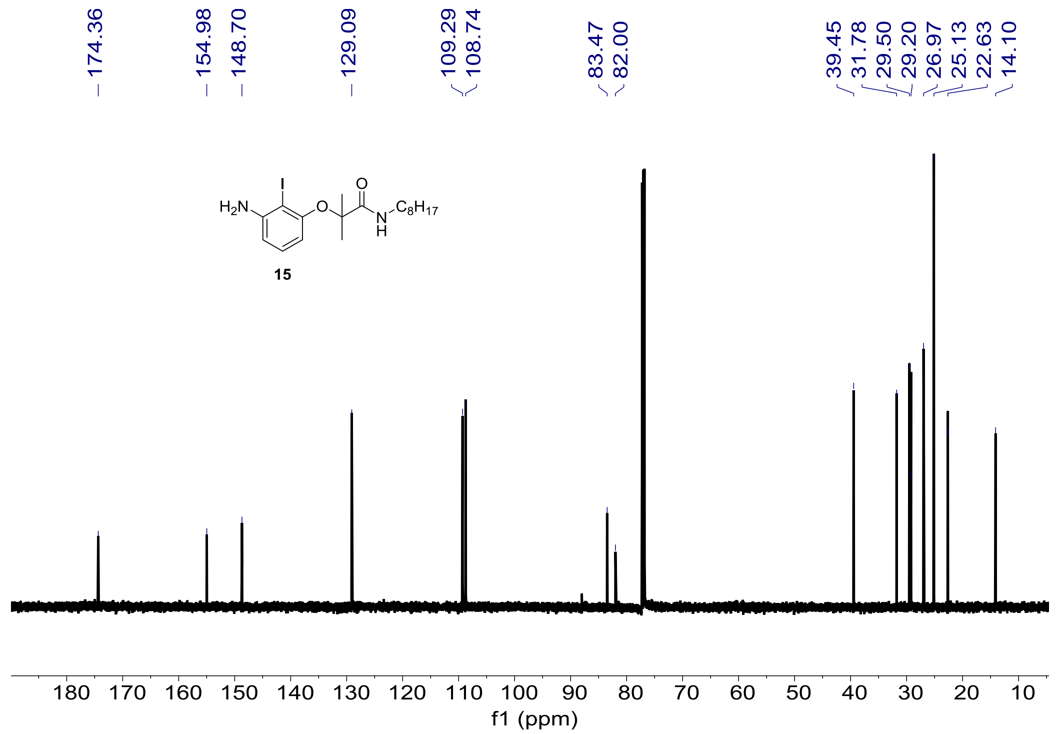

Supplementary Fig. 30 ^{13}C NMR (150 MHz) spectrum of **15** measured in $CDCl_3$ at 25 °C.

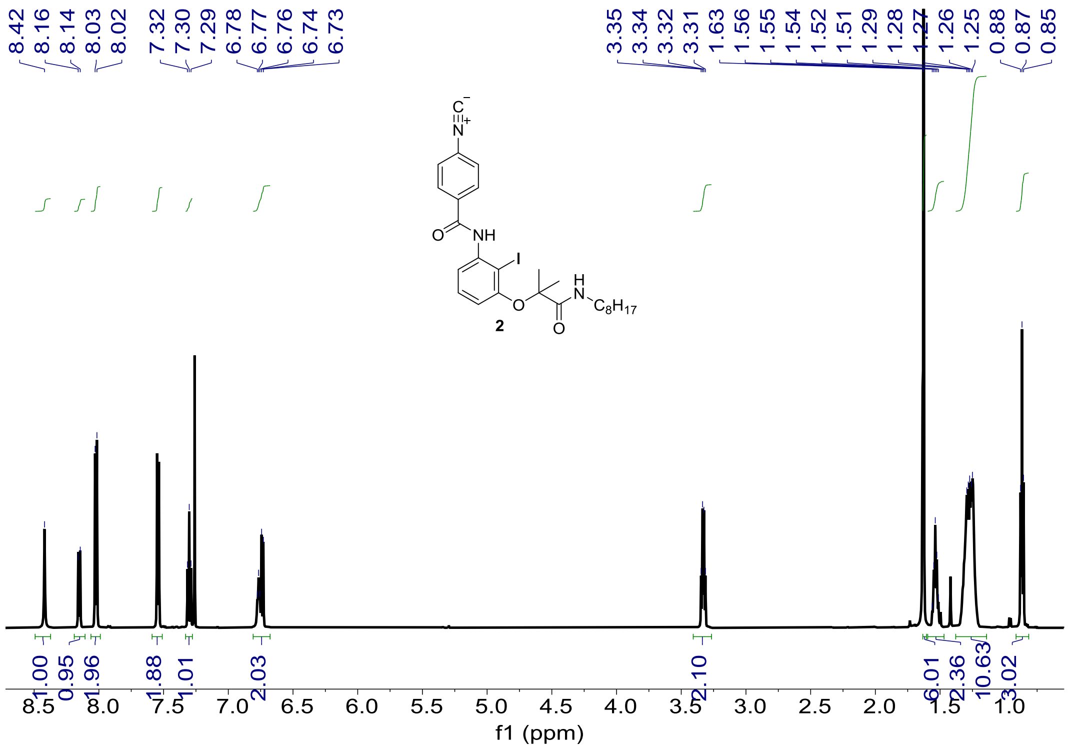

Supplementary Fig. 31 ^{1}H NMR (600 MHz) spectrum of **2** measured in $CDCl_3$ at 25 °C.

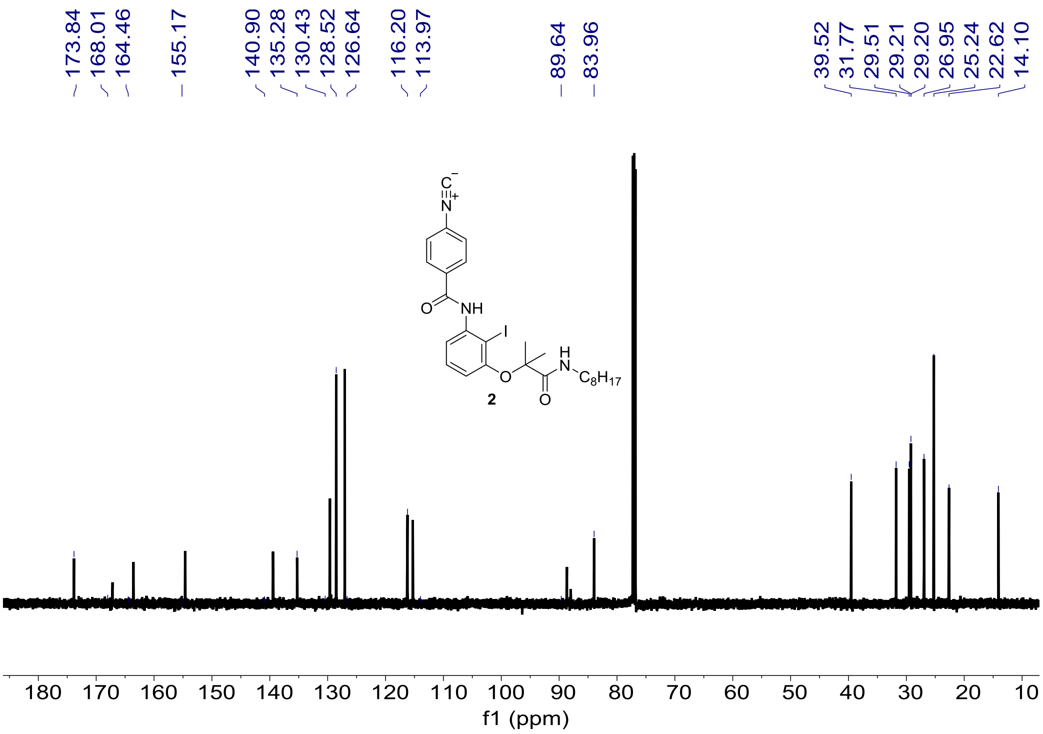

Supplementary Fig. 32 ^{13}C NMR (150 MHz) spectrum of **2** measured in $CDCl_3$ at 25 °C.

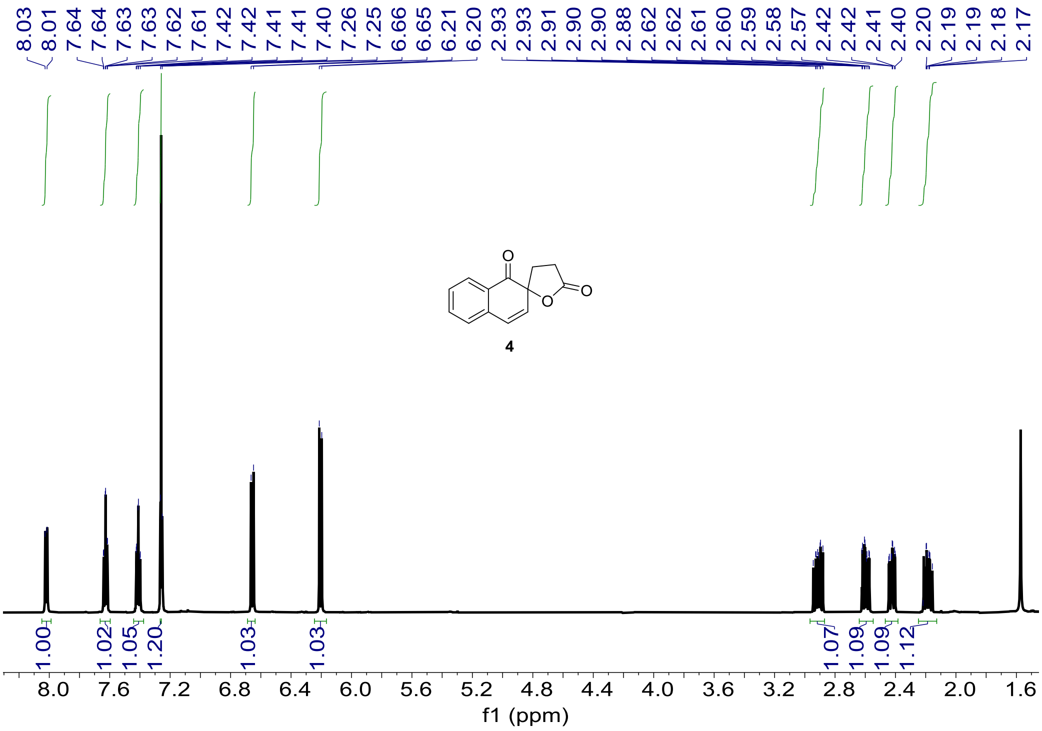

Supplementary Fig. 33 ^{1}H NMR (600 MHz) spectrum of **4** measured in $CDCl_3$ at 25 °C.

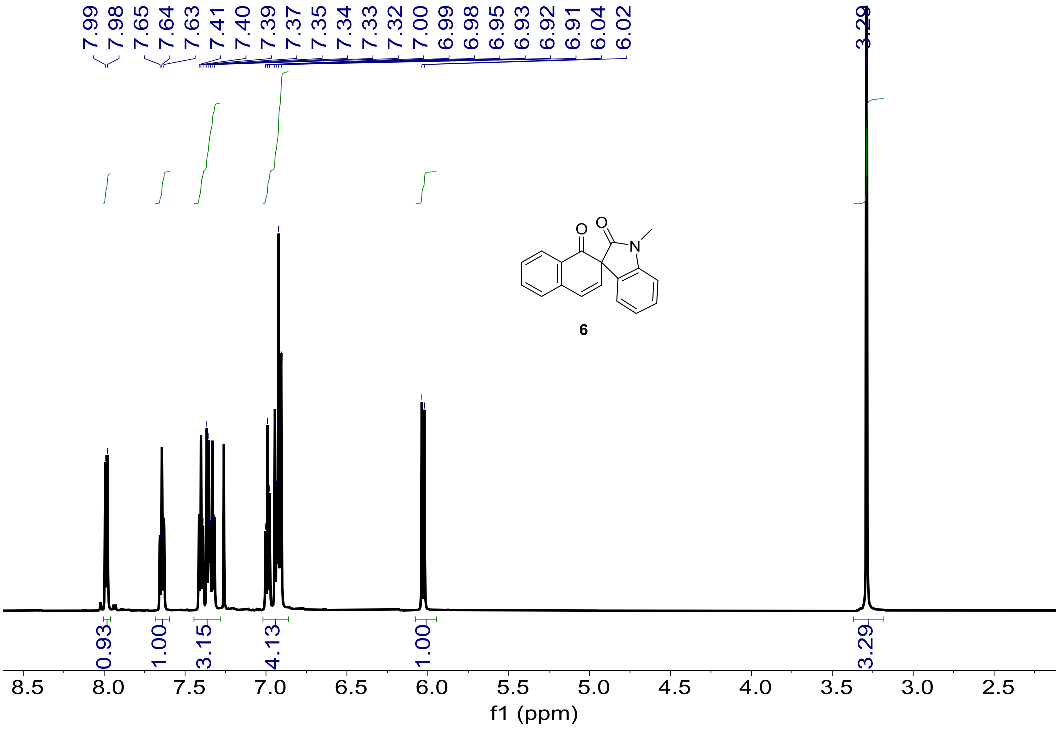

Supplementary Fig. 34 ^{1}H NMR (600 MHz) spectrum of **6** measured in $CDCl_3$ at 25 °C.

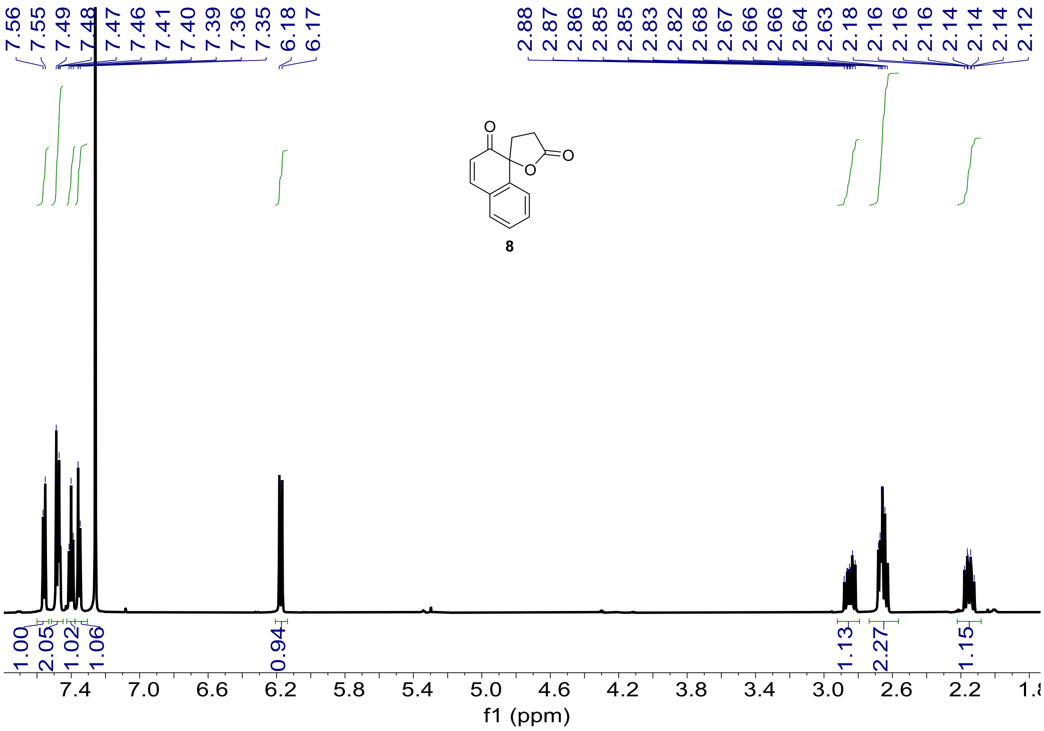

Supplementary Fig. 35 ^{1}H NMR (600 MHz) spectrum of **8** measured in $CDCl_3$ at 25 °C.

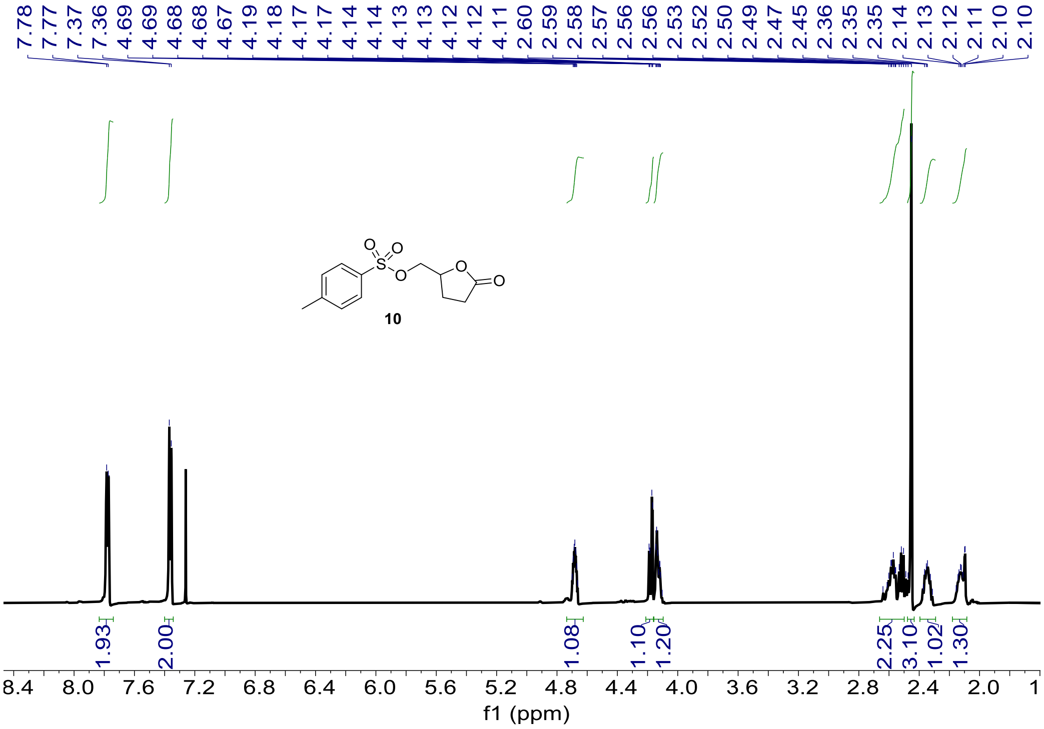

Supplementary Fig. 36 ^{1}H NMR (600 MHz) spectrum of **10** measured in $CDCl_3$ at 25 °C.

HPLC of the products

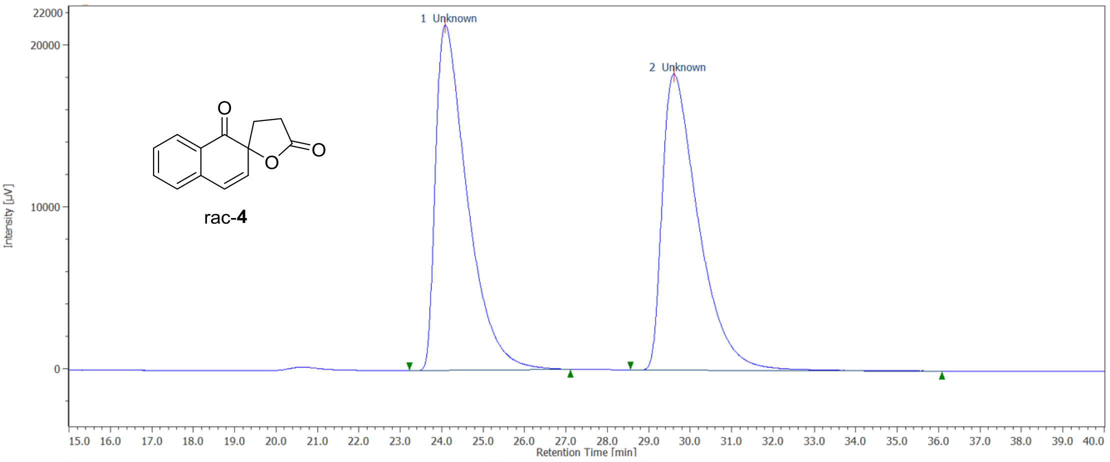

#	Peak Name	tR	Area	Height	Area%	Height%	Peak Start	Peak End	Base Start	Base End	Peak Mark
1	Unknown	24.083	1138047	21306	50.603	53.813	23.217	27.100	23.217	27.100	Manual
2	Unknown	29.600	1110920	18286	49.397	46.187	28.550	36.067	28.550	36.067	Manual

Supplementary Fig. 37 HPLC curve of racemic **4** catalyzed by poly-**2**$_{20}$ (Chiralpak OD-H; *n*-hexane/*i*-PrOH = 85/15 (v/v); 1.00 mL/min; 275 nm; 25 °C).

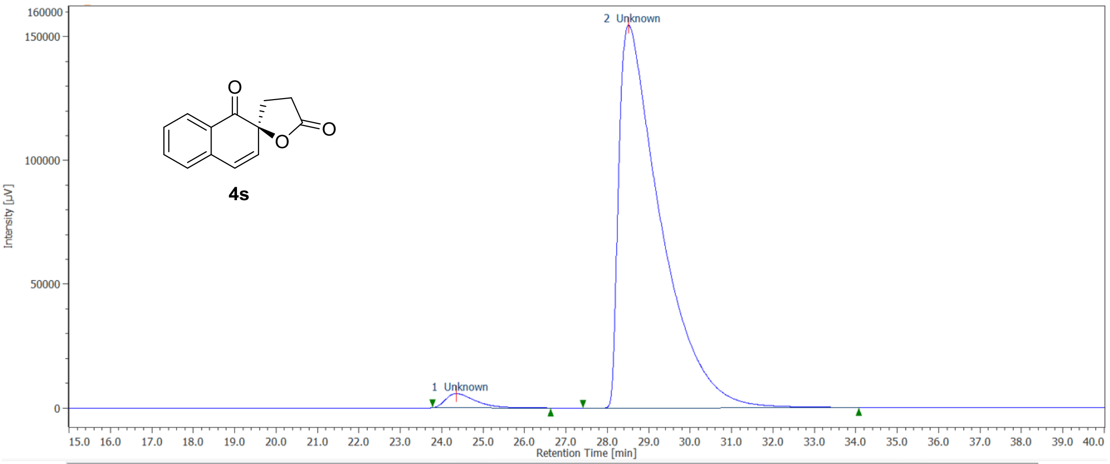

#	Peak Name	tR	Area	Height	Area%	Height%	Peak Start	Peak End	Base Start	Base End	Peak Mark
1	Unknown	24.350	282084	5720	2.539	3.572	23.775	26.625	23.775	26.625	Manual
2	Unknown	28.508	10826158	154396	97.461	96.428	27.408	34.058	27.408	34.058	Manual

Supplementary Fig. 38 HPLC curve of **4s** catalyzed by M-poly(L-**1**$_{150}$-*b*-**2**$_{10}$) (Chiralpak OD-H; *n*-hexane/*i*-PrOH = 85/15 (v/v); 1.00 mL/min; 275 nm; 25 °C).

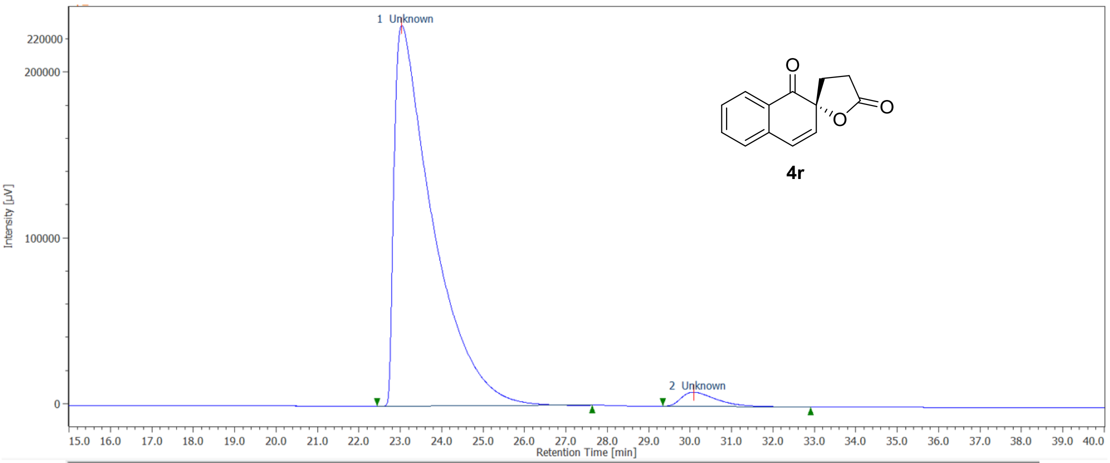

#	Peak Name	tR	Area	Height	Area%	Height%	Peak Start	Peak End	Base Start	Base End	Peak Mark
1	Unknown	23.033	14848794	229093	96.709	96.376	22.433	27.625	22.433	27.625	Manual
2	Unknown	30.075	505369	8614	3.291	3.624	29.333	32.900	29.333	32.900	Manual

Supplementary Fig. 39 HPLC curve of **4r** catalyzed by P-poly(L-**1**$_{50}$-*b*-**2**$_{10}$) (Chiralpak OD-H; *n*-hexane/*i*-PrOH = 85/15 (v/v); 1.00 mL/min; 275 nm; 25 °C).

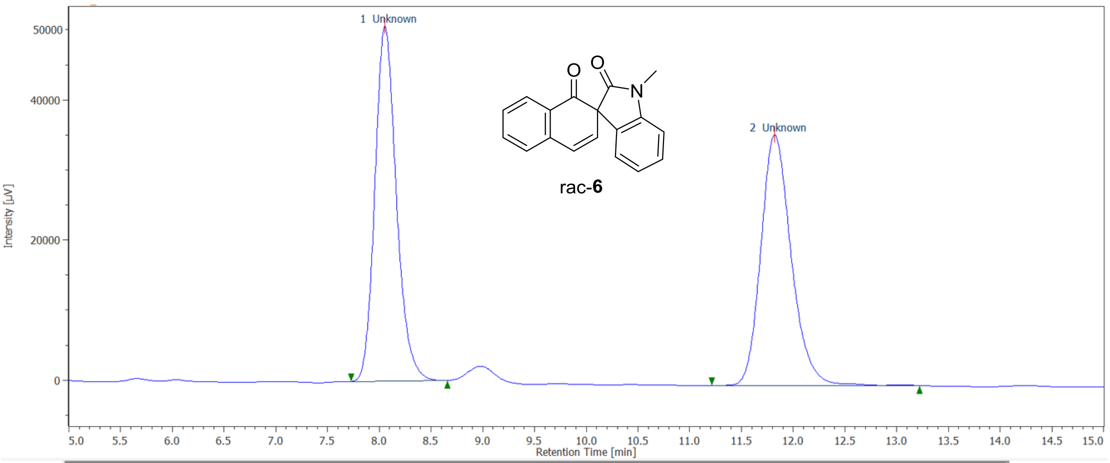

#	Peak Name	tR	Area	Height	Area%	Height%	Peak Start	Peak End	Base Start	Base End	Peak Mark
1	Unknown	8.050	730202	50669	50.054	58.612	7.725	8.658	7.725	8.658	Manual
2	Unknown	11.817	728635	35779	49.946	41.388	11.208	13.217	11.208	13.217	Manual

Supplementary Fig. 40 HPLC curve of racemic **6** catalyzed by poly-$\mathbf{2}_{20}$ (Chiralpak AD-H; *n*-hexane/*i*-PrOH = 70/30 (v/v); 1.00 mL/min; 254 nm; 25 °C).

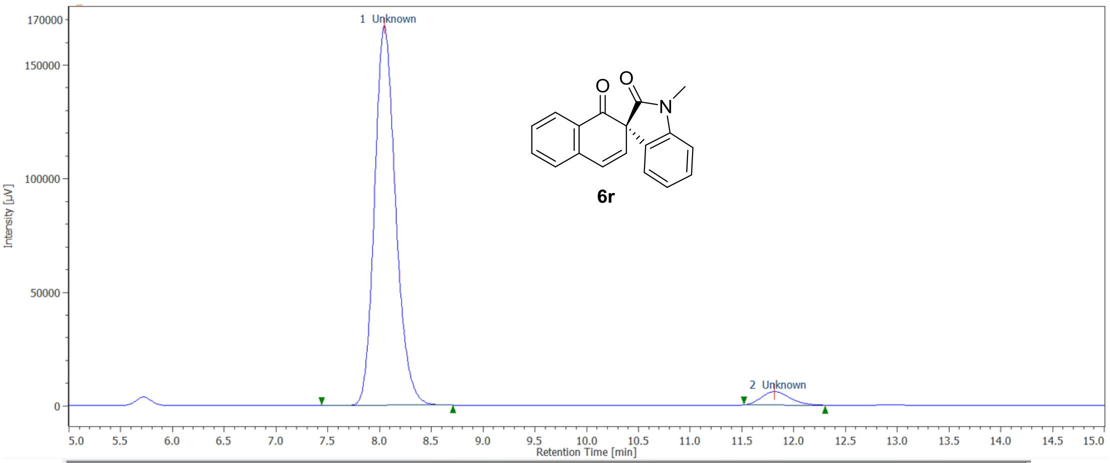

#	Peak Name	tR	Area	Height	Area%	Height%	Peak Start	Peak End	Base Start	Base End	Peak Mark
1	Unknown	8.042	2366690	166856	95.492	96.605	7.442	8.708	7.442	8.708	Manual
2	Unknown	11.808	111723	5864	4.508	3.395	11.517	12.300	11.517	12.300	Manual

Supplementary Fig. 41 HPLC curve of **6r** catalyzed by *M*-poly(L-$\mathbf{1}_{150}$-*b*-$\mathbf{2}_{10}$) (Chiralpak AD-H; *n*-hexane/*i*-PrOH = 70/30 (v/v); 1.00 mL/min; 254 nm; 25 °C).

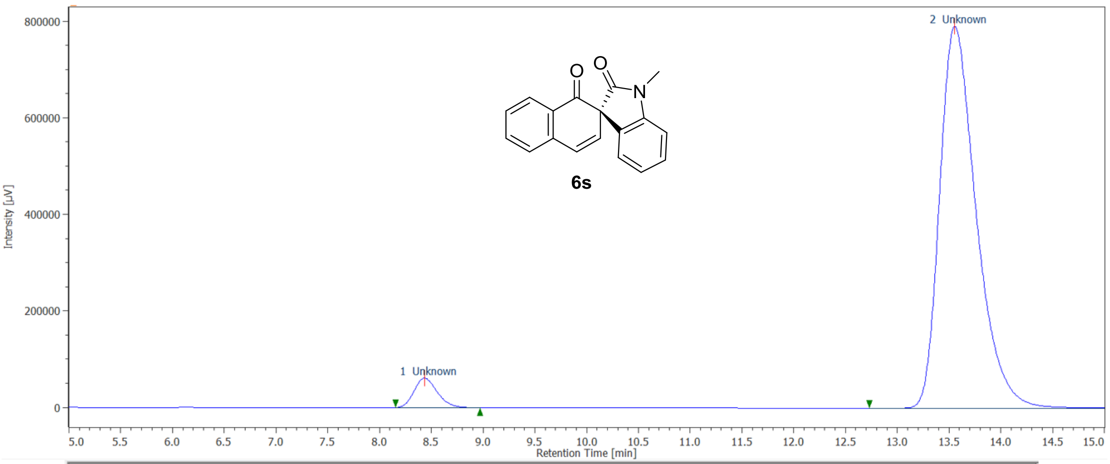

#	Peak Name	tR	Area	Height	Area%	Height%	Peak Start	Peak End	Base Start	Base End	Peak Mark
1	Unknown	8.433	960351	61424	4.732	7.213	8.150	8.967	8.150	8.967	Manual
2	Unknown	13.550	19333417	790178	95.268	92.787	12.725	15.283	12.725	15.283	Manual

Supplementary Fig. 42 HPLC curve of **6s** catalyzed by *P*-poly(L-$\mathbf{1}_{50}$-*b*-$\mathbf{2}_{10}$) (Chiralpak AD-H; *n*-hexane/*i*-PrOH = 70/30 (v/v); 1.00 mL/min; 254 nm; 25 °C).

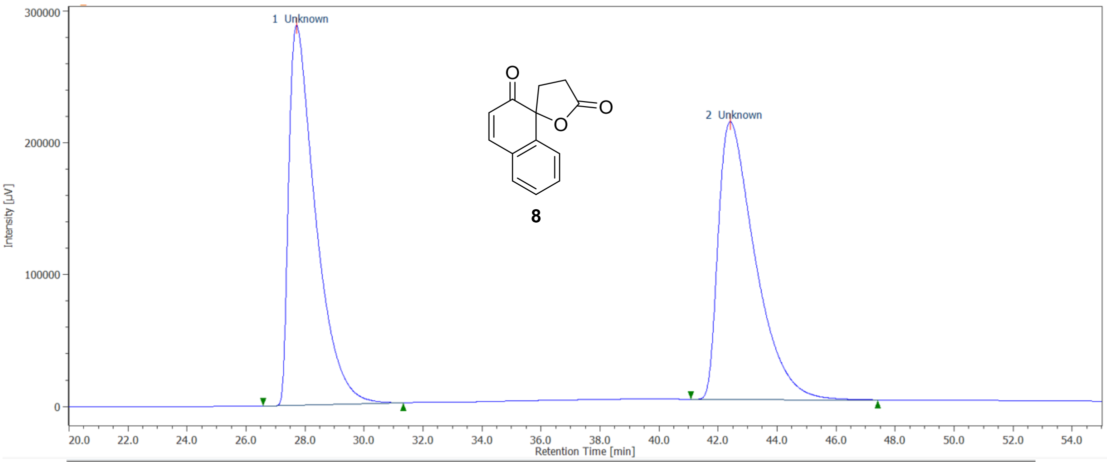

#	Peak Name	tR	Area	Height	Area%	Height%	Peak Start	Peak End	Base Start	Base End	Peak Mark
1	Unknown	27.708	18145859	287440	49.761	57.742	26.583	31.325	26.583	31.325	Manual
2	Unknown	42.400	18319816	210363	50.239	42.258	41.058	47.400	41.058	47.400	Manual

Supplementary Fig. 43 HPLC curve of racemic **8** catalyzed by poly-$\mathbf{2}_{20}$ (Chiralpak OD-H; *n*-hexane/*i*-PrOH = 85/15 (v/v); 1.00 mL/min; 230 nm; 25 °C).

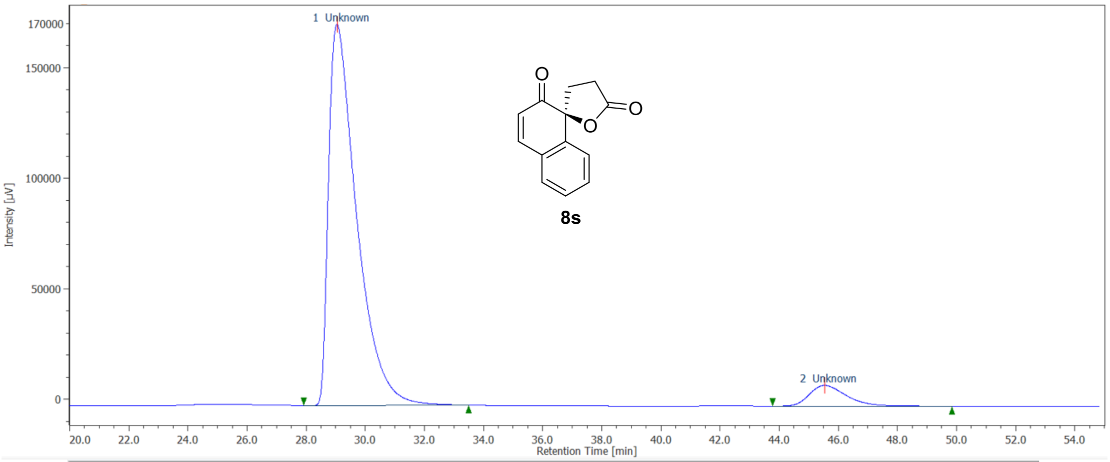

#	Peak Name	tR	Area	Height	Area%	Height%	Peak Start	Peak End	Base Start	Base End	Peak Mark
1	Unknown	29.042	11368655	172291	93.298	94.841	27.917	33.483	27.917	33.483	Manual
2	Unknown	45.525	816701	9372	6.702	5.159	43.767	49.825	43.767	49.825	Manual

Supplementary Fig. 44 HPLC curve of **8s** catalyzed by *M*-poly(L-$\mathbf{1}_{150}$-*b*-$\mathbf{2}_{10}$) (Chiralpak OD-H; *n*-hexane/*i*-PrOH = 85/15 (v/v); 1.00 mL/min; 230 nm; 25 °C).

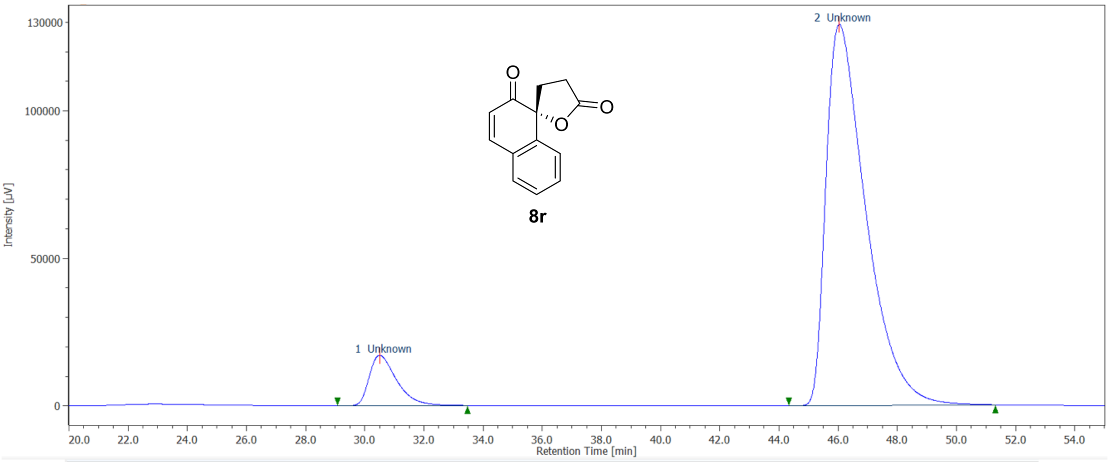

#	Peak Name	tR	Area	Height	Area%	Height%	Peak Start	Peak End	Base Start	Base End	Peak Mark
1	Unknown	30.492	1097135	17039	8.320	11.673	29.075	33.467	29.075	33.467	Manual
2	Unknown	46.025	12089395	128926	91.680	88.327	44.325	51.300	44.325	51.300	Manual

Supplementary Fig. 45 HPLC curve of **8r** catalyzed by *P*-poly(L-$\mathbf{1}_{50}$-*b*-$\mathbf{2}_{10}$) (Chiralpak OD-H; *n*-hexane/*i*-PrOH = 85/15 (v/v); 1.00 mL/min; 230 nm; 25 °C).

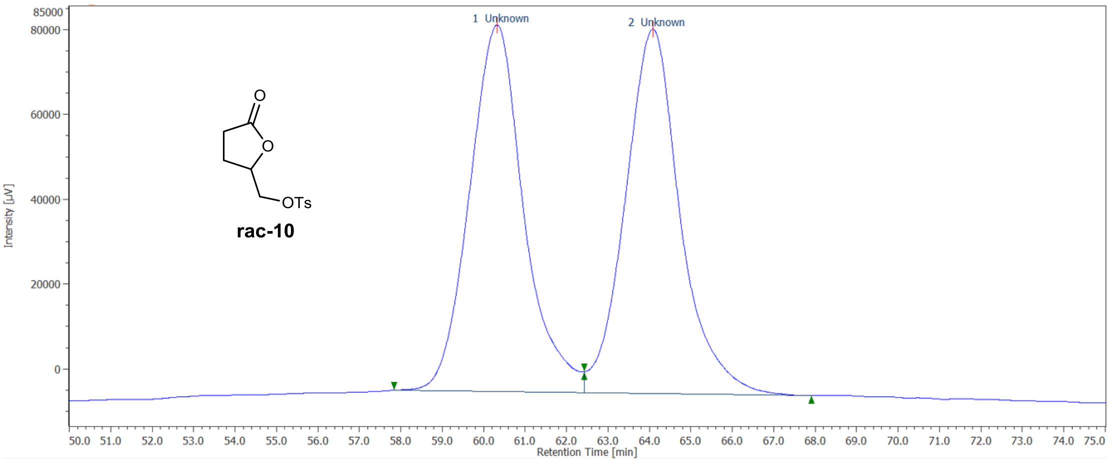

#	Peak Name	tR	Area	Height	Area%	Height%	Peak Start	Peak End	Base Start	Base End	Peak Mark
1	Unknown	60.317	7862452	86309	49.866	50.149	57.842	62.425	57.842	67.900	Manual
2	Unknown	64.075	7904604	85795	50.134	49.851	62.425	67.900	57.842	67.900	Manual

Supplementary Fig. 46 HPLC curve of racemic **10** catalyzed by poly-**2**$_{20}$ (Chiralpak AD-H; *n*-hexane/*i*-PrOH = 90/10 (v/v); 1.00 mL/min; 214 nm; 25 °C).

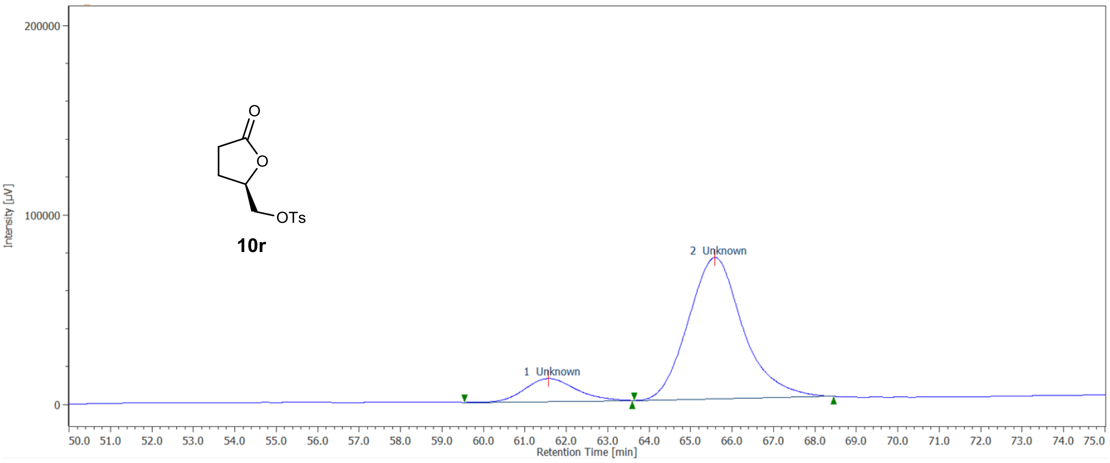

#	Peak Name	tR	Area	Height	Area%	Height%	Peak Start	Peak End	Base Start	Base End	Peak Mark
1	Unknown	61.567	1023939	12088	13.014	13.962	59.533	63.575	59.533	63.575	Manual
2	Unknown	65.575	6844110	74495	86.986	86.038	63.633	68.442	63.633	68.442	Manual

Supplementary Fig. 47 HPLC curve of **10r** catalyzed by *M*-poly(L-**1**$_{150}$-*b*-**2**$_{10}$) (Chiralpak AD-H; *n*-hexane/*i*-PrOH = 90/10 (v/v); 1.00 mL/min; 214 nm; 25 °C).

Supplementary References

1. Chen, J.-L., Yang, L., Wang, Q., Jiang, Z.-Q., Liu, N., Yin, J., Ding, Y.-S., Wu, Z.-Q. Helix-Sense-Selective and Enantiomer-Selective Living Polymerization of Phenyl Isocyanide Induced by Reusable Chiral Lactide Using Achiral Palladium Initiator. *Macromolecules* **48**, 7737–7746 (2015).

2. Yin, J., Xu, L., Han, X., Zhou, L., Li, C., Wu, Z.-Q. A facile synthetic route to stereoregular helical poly(phenyl isocyanide)s with defined pendants and controlled helicity. *Polym. Chem.* **8**, 545–556 (2017).
3. Xue, Y.-X., Zhu, Y.-Y., Gao, L.-M., He, X.-Y., Liu, N., Zhang, W.-Y., Yin, J., Ding, Y., Zhou, H., Wu, Z.-Q. Air-Stable (Phenylbuta-1,3-diynyl)palladium(II) Complexes: Highly Active Initiators for Living Polymerization of Isocyanides. *J. Am. Chem. Soc.* **136**, 4706–4713 (2014).
4. Wu, Z.-Q., Nagai, K., Banno, M., Okoshi, K., Onitsuka, K., Yashima, E. Enantiomer-Selective and Helix-Sense-Selective Living Block Copolymerization of Isocyanide Enantiomers Initiated by Single-Handed Helical Poly(phenyl isocyanide)s. *J. Am. Chem. Soc.* **131**, 6708–6718 (2009).
5. He, T., Peng, L., Li, S., Hu, F., Xie, C., Huang, S., Jia, S., Qin, W., Yan, H. Chiral Naphthyl-C2-Indole as Scaffold for Phosphine Organocatalysis: Application in Asymmetric Formal [4 + 2] Cycloaddition Reactions. *Org. Lett.* **22**, 6966−6971 (2020).
6. Kevin, A., Laurent, P., Denis, D., Stephane, M., Philippe, A.-P., Stephane, Q. Synthesis of [7] Helicene Enantiomers and Exploratory Study of Their Conversion into Helically Chiral Iodoarenes and Iodanes. *Chem. Eur. J.* **25**, 2852–2858 (2019).
7. Zhang, D.-Y., Xu, L., Wu, H., Gong, L.-Z. Chiral Iodine-Catalyzed Dearomatizative Spirocyclization for the Enantioselective Construction of an All-Carbon Stereogenic Center. *Chem. Eur. J.* **21**, 10314–10317 (2015).
8. Uyanik, M., Yasui, T., Ishihara, K. Chiral Hypervalent Organoiodine-Catalyzed EnantioselectiveOxidative Spirolactonization of Naphthol Derivatives. *J. Org. Chem.* **82**, 11946−11953 (2017).
9. Gelis, C., Dumoulin, A., Bekkaye, M., Neuville, L., Masson, G. Chiral Hypervalent Iodine(III) Catalyst Promotes Highly Enantioselective Sulfonyl- and Phosphoryl-oxylactonizations. *Org. Lett.* **19**, 278−281 (2017).
